# Efficacy and safety of Shenfu injection for the treatment of post-acute myocardial infarction heart failure: A systematic review and meta-analysis

**DOI:** 10.3389/fphar.2022.1027131

**Published:** 2022-11-24

**Authors:** Yanhua Wu, Shuang Li, Zunjiang Li, Zhaofan Mo, Ziqing Luo, Dongli Li, Dawei Wang, Wei Zhu, Banghan Ding

**Affiliations:** ^1^ The Second Affiliated Hospital of Guangzhou University of Chinese Medicine, Guangzhou, China; ^2^ Guangdong Provincial Hospital of Chinese Medicine, Guangzhou, China; ^3^ State Key Laboratory of Emergency of Chinese Medicine, Guangdong Provincial Hospital of Chinese Medicine, Guangzhou, China; ^4^ The First Clinical College of Guangzhou University of Chinese Medicine, Guangzhou, China; ^5^ The Second Clinical College of Guangzhou University of Chinese Medicine, Guangzhou, China; ^6^ Animal Experiment Centre of Guangzhou University of Chinese Medicine, Guangzhou, China; ^7^ Shunde Hospital of Guangzhou University of Chinese Medicine, Guangzhou, China

**Keywords:** acute myocardial infarction, heart failure, Shenfu injection, meta-analysis, systematic review, traditional Chinese medicine

## Abstract

**Objective:** This systematic review and meta-analysis aimed to investigate the adjuvant effect and safety of Shenfu injection (SFI) on the treatment of post-acute myocardial infarction heart failure (PAMIHF).

**Methods:** Seven databases were searched to identify randomized controlled trials (RCTs) associated with SFI and PAMIHF treatment from May 1990 to May 2022. Primary outcomes included NT-proBNP and left ventricular ejection fraction (LVEF), and secondary outcomes included total effective rate, BNP, heart rate (HR), cardiac output (CO), and adverse event (AE). The risk of bias evaluation was assessed by the ROB2 tool, meta-analysis, subgroup analysis, sensitivity analysis, and publication bias were conducted by RevMan5.3 software, and the Grade of Recommendations, Assessment, Development, and Evaluations (GRADE) system was used to evaluate the quality of evidence of meta results.

**Results:** A total of 36 studies with 3231 PAMIHF patients were included. The meta results suggested that adjuvant SFI therapy was superior to conventional medical therapy alone. It improved the total effective rate [RR = 1.33; 95% CI (1.25.1.40); *p* < 0.00001], increased LVEF [SMD = 0.98; 95% CI (0.71, 1.24); *p* < 0.00001], and decreased HR [SMD = −1.14; 95% CI (−1.28, −0.99); *p* < 0.00001]. In addition, adjuvant SFI therapy (9.73%, 66/678) had a rate of AE lower than that of conventional medical therapy alone (21.7%, 147/677) when regarding safety [RR = 0.45; 95% CI (0.35, 0.57); *p* < 0.00001]. The quality of the evidence for the outcomes was rated from “very low” to “moderate.”

**Conclusion:** Adjuvant SFI therapy was safer to improve the total effective rate and the heart function of PAMIHF patients. However, well-designed RCTs were needed to confirm the efficacy and safety of adjuvant SFI therapy in PAMIHF treatment due to the low quality of the evidence for the outcomes caused by a small sample size and unclear risk of bias existed in included studies.

**Systematic Review Registration:** https://www.crd.york.ac.uk/PROSPERO/display_record.php?RecordID=151856), identifier CRD42020151856.

## 1 Introduction

Acute myocardial infarction (AMI) is a clinical syndrome, and it is mainly characterized by chest pain, shortness of breath, sweating, and abnormal heart beating, due to sudden reduction of blood flow and imbalance between myocardial oxygen supply and demand (Sandoval and Jaffe, 2019). Heart failure (HF) is a syndrome mainly associated with systematic congestion and ultimately organ dysfunction due to hypoperfusion (Arrigo et al., 2020). HF, a common complication of AMI, is the major driver of long-term mortality, high medical costs, and 3–6 times of risk of death within 30 days (Song and Jin, 2021). Despite the remarkable advances in AMI treatment over the past 2 decades, incidence of post-acute myocardial infarction heart failure (PAMIHF) among hospitalized patients remains high ranging from 14% to 36%. Thus, new and alternative medical management of PAMIHF remains challenging and urgently needed (Bahit et al., 2018).

Shenfu injection (SFI) is a traditional Chinese medical formulation, and it is prepared from *Panax ginseng* C.A. Meyer (Araliaceae, *Ginseng radix et rhizoma*) and *Aconitμm carmichaelii Debx* (Ranunculaceae, *Aconiti radix*). 1ml of SFI is extracted from 0.1 g of *Panax ginseng* C.A. Meyer and 0.2 g of *Aconitμm carmichaelii Debx.* (Wang et al., 2021a). The main active ingredients of SFI were identified as ginsenosides and aconite alkaloids by combinatory liquid chromatography–mass spectrometric techniques (Wang et al., 2021a). SFI has been widely used in the treatment of cardiovascular and cerebrovascular diseases, especially in HF treatment (Su et al., 2018). It has the functions of improving organ perfusion, protecting myocardium and tissue damage during cerebral ischemia (Li et al., 2013), improving hemodynamics, dilating blood vessels (Li, 2017), anti-inflammatory effects (Li et al., 2019), etc. However, it still lacks evaluation on the efficacy and safety of SFI in the treatment of PAMIHF in terms of methodology and quality of evidence.

In this study, we aimed to elucidate the efficacy and safety of SFI as an adjunctive treatment for AMI-HF through the available evidence in practice. We mainly focused on clarifying whether SFI combined with conventional therapy had an adjuvant effect compared with conventional therapy alone and was as safe as conventional therapy.

## 2 Data and methods

### 2.1 The composition of SFI

Shenfu injection, comprising *Panax ginseng* C.A. Meyer (Araliaceae, *Ginseng radix et rhizoma*) and *Aconitμm carmichaelii Debx* (Ranunculaceae, *Aconiti radix*), is derived from the traditional Chinese medicine formula Shenfu decoction, which has been used in China for over hundreds of years. Several studies have reported the chemical profile of SFI using different methods; SFI mainly includes *Aconitine alkaloids*, *Ginsenosaponin*, *Aconitum alkaloids*, *Ginsenoside*, *Aconitine*, and *Hydrophilic compound* (for details, see Supplementary Table S1), among which ginsenosides and aconite alkaloids are identified as the main active ingredients of SFI (Yang et al., 2014; Gao et al., 2016; Li et al., 2016).

### 2.2 Database for search

Here, three English databases (MEDLINE *via* PubMed, EMBASE, and Web of science) and four Chinese databases [China National Knowledge Infrastructure (CNKI), WanFang Database, Chinese Biomedical Literature Database (CBM), and China Science and Technology Journal Database (VIP)] were searched from May 1990 to May 2022.

### 2.3 Criteria for studies included

#### 2.3.1 Type of participants (P)

Patients aged more than 18 years who were diagnosed with AMI and HF according to the diagnostic criteria recognized in certain guidelines, literature, or certain books were included, regardless of nationality, gender, race, age, course of disease, and types of heart failure, STEMI NSTEMI, HFrEF, or HFpEF.

#### 2.3.2 Type of interventions (I and C)

Control group: Conventional western medicine treatment, including low-salt diet, lipid lowering, vasodilator, diuretic, cardiotonic, oxygen inhalation, and restriction of fluid intake. Experimental group: SFI treatment plus the control group.

#### 2.3.3 Type of outcome measures

Primary outcomes (O): ① Left ventricular ejection fraction (LVEF) and ② NT-proBNP; secondary outcomes: ① Total clinical effective rate (for definition, see Supplementary file S2), ② heart rate (HR), ③ cardiac output (CO), and ④ BNP; safety outcome: Adverse events.

#### 2.3.4 Types of studies (S)

The studies were randomized controlled trials (RCTs).

### 2.4 Exclusion criteria

The exclusion criteria are as follows: ① repeated publications, ② pure theoretical research, ③ case report, and ④ not complete data.

### 2.5 Searching strategy

We searched studies with [Title/Abstract] by developing the search strategies of the combination of the MeSH terms (participants, intervention, comparison, outcomes, and study design), including P+1, P + I + C, P + I + C + O, and P + I + C + O + S. If the number of studies retrieved was small, we searched by P + I and then manually screened studies based on inclusion and exclusion criteria.

### 2.6 Data collection and analysis

#### 2.6.1 Selection of studies

Two review authors independently screened titles and abstracts of studies identified by literature search according to the criteria of PICOS. Duplication was omitted using NoteExpress software. Then, another two authors extracted and summarized the data from the included studies. Discrepancies were resolved by consensus.

#### 2.6.2 Data extraction and management

The details of studies were identified separately by two reviewers and were presented in a standardized table. Two authors independently extracted the data including the sample size, age, treatment details, criteria for AMI and AHF diagnosis, outcomes, and adverse events.

#### 2.6.3 Evaluation of risk of bias

Two authors independently evaluated the methodological quality of the screened studies by using the ROB2 tool according to the instructions (https://methods.cochrane.org/bias/resources/rob-2-revised-cochrane-risk-bias-tool-randomized-trials). The specific criteria for risk of bias mainly included the following five aspects: randomization process, deviations from the intended interventions, missing outcome data, measurement of the outcome, and selection of the reported result. Quality assessments were rated as “high risk,” “some concerns,” or “unclear” risk of bias. All the authors discussed to address any discrepancies.

#### 2.6.4 Data synthesis and analysis

The Review Manager Software tool (RevMan, v.5.3; The Cochrane Collaboration) was used to synthesize the data. We pooled the mean differences for dichotomous data with relative risk (RR) and 95% confidence intervals (CIs), while continuous data were pooled with standard mean difference (SMD) and 95% CI. When I^2^ ≤ 75%, we used the fixed-effects model to synthesize the data. When I^2^>75%, we used the random-effects model to synthesize the data.

#### 2.6.5 Sensitivity analysis

We aim to assess whether the conclusions were robust for the decision-making process, and we explored significant heterogeneity between studies by sensitivity analysis. When the analysis showed high heterogeneity, we performed a sensitivity analysis by removing a single study to observe whether the new effect size results and heterogeneity changed significantly.

### 2.7 Evidence confidence

The certainty of evidence was assessed by using the Graded Recommendation Assessment, Development, and Evaluations (GRADE) technique (https://www.gradepro.org/) according to risk of bias, indirectness, inconsistency, imprecision, and publication bias. The level of evidence was classified as high, moderate, low, or very low.

## 3 Results

### 3.1 Results of RCT selection

**FIGURE 1:**
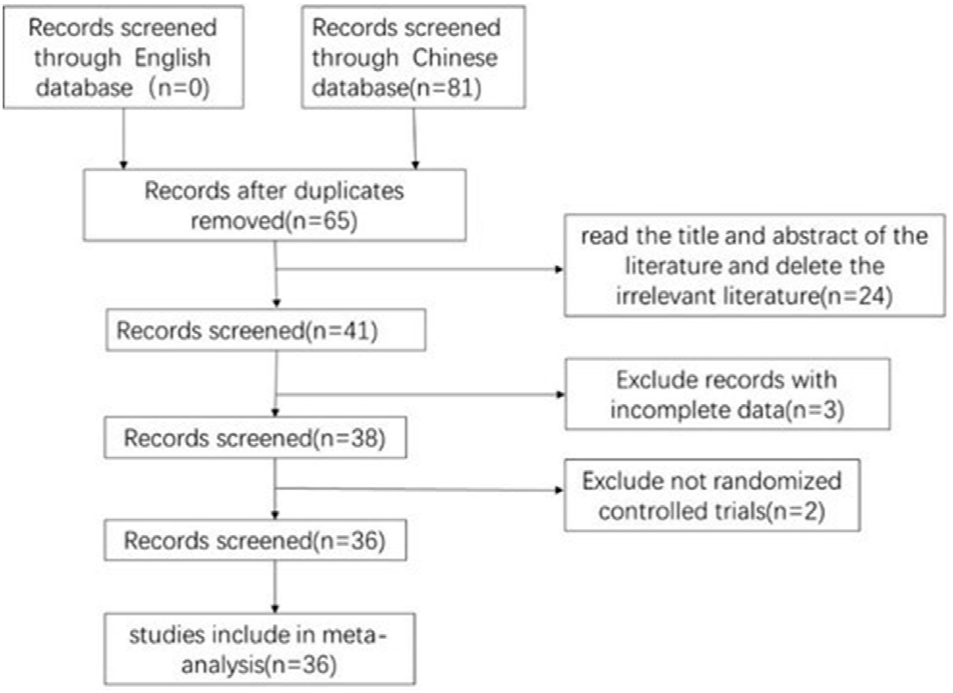
PRISMA flow diagram of the literature screening and selection process.

A total of 147 related articles were initially detected. After excluding 82 replicate studies, 65 RCTs were included for further screening. After a detailed reading of the article titles and abstracts, 24 irrelevant studies, 3 studies with incomplete data, and 2 non-RCT studies were excluded. Finally, 36 studies were included (Mo and Zhao, 2002; Song et al., 2002; Zeng, 2005; Li et al., 2006; Guo et al., 2010; Li et al., 2010; Zhang, 2011; Zhi-Qing et al., 2011; Zhang et al., 2012; Guo et al., 2013; Zou, 2013; Guo, 2014; He and Sheng, 2014; Meng, 2014; Zong et al., 2014; Li, 2015; Xu et al., 2015; Li, 2016; Li and Chen, 2016; Sun, 2016; Zhao et al., 2016; Li and Hou, 2017; Wang and Qin, 2017; Wang et al., 2017; Yan et al., 2017; Wang, 2018; Wang et al., 2018; Zhang et al., 2018; Zhang and Zhang, 2018; Zhao, 2018; Fen et al., 2019; You and Wang, 2019; Zhang et al., 2019; Wang, 2020; Zhang, 2020; Wang, 2021), with a total of 3231 patients with PAMIHF for the systematic review and meta-analysis. Figure 1 describes the literature screening process and results, and details for search results are supplied in Supplementary File S1.

### 3.2 Characteristics of included RCTs

A total of 36 RCTs were conducted in China from 2002 to 2021, with sample sizes ranging from 46 to 334 and treatment durations ranging from 5 to 28 days, except for one study (Zou, 2013) that did not report the sustained time. In addition to two studies (Guo et al., 2013; Zhang et al., 2019) that did not mention the age, the mean age ranged from 46 to 76 years in other studies. In terms of the usage and dosage of SFI, three studies (Li et al., 2010; Zou, 2013; Zhang et al., 2018) did not report the dosage; the dosage of other studies varied from 20 to 100 ml. All the studies reported that SFI was diluted with 250–500 ml 5% dextrose, 100–500 ml 0.9% saline, or direct intravenous injection. Also, two studies (Zou, 2013; Zhang et al., 2018) did not record the usage, and one study (Wang and Qin, 2017) used the pump method. Moreover, eight studies (Zhi-Qing et al., 2011; Guo et al., 2013; Zou, 2013; Guo, 2014; Xu et al., 2015; Wang et al., 2017; Zhang et al., 2018; Zhang, 2020) did not report diagnostic criteria for AMI, and nine studies (Zhi-Qing et al., 2011; Guo et al., 2013; Zou, 2013; Guo, 2014; Xu et al., 2015; Sun, 2016; Wang et al., 2017; Zhang et al., 2018; Zhang, 2020) did not report diagnostic criteria for HF. The diagnostic criteria for AMI in one study (Li et al., 2006) were consistent with the literature (Sun and Fu, 2004). The diagnostic criteria for AMI in 15 studies (Mo and Zhao, 2002; Song et al., 2002; Zeng, 2005; Zhang, 2011; Zhang et al., 2012; Meng, 2014; Zong et al., 2014; Li and Chen, 2016; Sun, 2016; Zhao et al., 2016; Li and Hou, 2017; Wang, 2018; Wang et al., 2018; Fen et al., 2019; You and Wang, 2019) and the diagnostic criteria for HF in 23 studies (Mo and Zhao, 2002; Song et al., 2002; Zeng, 2005; Li et al., 2006; Guo et al., 2010; Zhang, 2011; Zhang et al., 2012; He and Sheng, 2014; Meng, 2014; Zong et al., 2014; Li, 2015; Li, 2016; Li and Chen, 2016; Zhao et al., 2016; Li and Hou, 2017; Wang and Qin, 2017; Yan et al., 2017; Wang, 2018; Wang et al., 2018; You and Wang, 2019; Zhang et al., 2019; Wang, 2020; Wang, 2021) met certain books (Chen, 1996; Wenwu, 2000; Chen, 2008). Diagnostic criteria for AMI in eight studies (Guo et al., 2010; Li et al., 2010; Li, 2015; Li, 2016; Wang and Qin, 2017; Yan et al., 2017; Zhang et al., 2019; Wang, 2020) were consistent with certain guidelines (Gao, 2001; Guidelines for the diagnosis and, 2010; Guidelines for the diagnosis and, 2015). Also, four studies (He and Sheng, 2014; Zhang and Zhang, 2018; Zhao, 2018; Wang, 2021) for AMI and two studies (Zhang and Zhang, 2018; Zhao, 2018) for HF had corresponding diagnostic criteria without mentioning the source of the reference. The diagnostic criteria for HF in two studies (Li et al., 2010; Fen et al., 2019) conformed to the NYHA classification, but did not mention the source of the relevant literature. A majority of patients in 11 studies (Mo and Zhao, 2002; Zeng, 2005; Zhang et al., 2012; Meng, 2014; Zong et al., 2014; Li, 2016; Sun, 2016; Zhao et al., 2016; Wang and Qin, 2017; Wang et al., 2018; Zhang and Zhang, 2018) received percutaneous coronary intervention (PCI). One study (Yan et al., 2017) mentioned that none of the patients received PCI treatment, and 24 studies did not record whether the patients received the PCI treatment or not. None of the studies reported the follow-up results. The essential characteristics of the included RCTs are listed in Table 1.

**TABLE 1:** Characteristics of included RCTs investigating the adjunctive effect of Shenfu injection (SFI) on acute myocardial infarction and heart failure.

Included study (author/year/language)	Sample size (E/C)	Average age (E/C)	Duration	Interventions		Usage and dose	AHF diagnostic criteria	Adverse events	Outcome
				Experiment group	Control group				
Fen et al. (2019)	174/160	60.79 ± 9.73/61.43 ± 7.22	10 days	SFI plus CWT + E	CWT + E	Diluted in 5% GS 250ml, IVGTT	a	NR	②⑤⑨
Guo et al. (2010)	35/35	65.2 ± 14.2/63.7 ± 13.6	14 days	SFI plus CWT + rt-PA	CWT + rt-PA	Diluted in 0.9% NS 100ml, IVGTT	d	Death	⑨⑪
Guo et al. (2013)	30/30	NR	14 days	SFI plus CWT	CWT	Diluted in 5% GS/0.9% NS, IVGTT	NR	NR	①
Guo (2014)	40/40	73.70 ± 16.2/71.20 ± 14.6	14 days	SFI plus CWT	CWT	Diluted in 5% GS 100ml, IVGTT	NR	NR	①⑦⑨
He and Sheng (2014)	45/45	62.10 ± 2.4/60.13 ± 3.11	14 days	SFI plus CWT	CWT	Diluted in 0.9% NS 100ml, IVGTT (2 times/d)	NR	Death	①②⑤⑥⑨⑪
Li et al. (2006)	37/36	63.7 ± 18.6/59.8 ± 17.2	14 days	SFI plus CWT	CWT	IVGTT	e	NR	⑨
Li et al. (2010)	58/34	68.2 ± 9.3/67.8 ± 10.7	14 days	SFI plus CWT	CWT	IVGTT	d	NR	②③⑧⑪
Li (2015)	32/32	63.50 ± 11.2/63.20 ± 11.50	14 days	SFI plus CWT	CWT	Diluted in 0.9% NS 100ml, IVGTT (2 times/d)	g	Death	①②⑤⑥⑨⑪
Li (2016)	32/32	62.73 ± 8.23/62.73 ± 8.23	<14 days	SFI plus CWT + D	CWT + D	IVGTT	g	Death	⑧
Li and Chen (2016)	23/23	68.60 ± 2.60/68.70 ± 2.60	14 days	SFI plus CWT	CWT	Diluted in 5% GS 200ml, IVGTT	i	Bleeding	①②⑥⑦⑨⑪
Li and Hou (2017)	31/31	66.38 ± 10.69/67.41 ± 11.98	14 days	SFI plus CWT	CWT	Diluted in 0.9% NS 100ml, IVGTT	d	Death, SMI, bleeding, blood clots, arrhythmia	①②⑥⑧⑪
Meng (2014)	30/30	46.3 ± 11.9/46.7 ± 12.1	5 days	SFI plus CWT + Du	CWT + Du	IVGTT	i	NR	①②⑦⑪
Mo and Zhao (2002)	36/38	55.3 ± 15.6/54.9 ± 12.7	7 days	SFI plus CWT	CWT	Diluted in 5% GS/0.9% NS 250ml, IVGTT	l	NR	①⑨
Song et al. (2002)	24/24	56.23 ± 4.53/54.81 ± 4.37	20 days	SFI plus CWT + Du	CWT + Du	Diluted in 5% GS 250ml, IVGTT	m	Tachycardia, Hypertension, Ventricular Premature	①②⑧
Sun (2016)	31/31	65.3 ± 5.1/67.1 ± 5.3	7 days	SFI plus CWT + rhBNP	CWT + rhBNP	Diluted in 5% GS 250ml, IVGTT	a	Low blood pressure	⑧⑩
Wang and Qin (2017)	64/64	59.7 ± 14.3/58.2 ± 13.6	7 days	SFI plus CWT + D	CWT + D	pump	g	Arrhythmia	①⑧⑪
Wang et al. (2017)	44/44	72.79 ± 10.56/72.09 ± 10.62	14 days	SFI plus CWT + Simvastatin	CWT + Simvastatin	IVGTT	NR	NR	②③⑤⑪
Wang (2018)	58/58	60.8 ± 2.5/64.8 ± 2.5	7 days	SFI plus rhBNP	rhBNP	Diluted in 5% GS 250–500ml, IVGTT	f	Low blood pressure, arrhythmia	②③
Wang et al. (2018)	31/31	64.8 ± 2.5/60.8 ± 2.5	7 days	SFI plus CWT + rhBNP	CWT + rhBNP	Diluted in 5% GS 250ml, IVGTT	NR	Low blood pressure, arrhythmia	②③⑤⑧⑩⑪
Wang (2020)	37/37	65.78 ± 5.52/65.13 ± 5.39	7 days	SFI plus Lyophilized rhBNP	Lyophilized rhBNP	Diluted in 5% GS 250ml, IVGTT	d	NR	②⑤
Wang (2021)	33/32	73 ± 12.8/72 ± 13.6	10 days	SFI plus CWT	CWT	Diluted in 5–10% GS 250–500ml, IVGTT	NR	NR	①②⑨
Zhi-Qing et al. (2011)	37/33	75.8 ± 12.3/74.3 ± 11.5	14 days	SFI plus CWT	CWT	Diluted in 5% GS/0.9% NS, IVGTT	NR	Low blood pressure, arrhythmia, infection	①②⑧⑪
Xu et al. (2015)	36/38	55.3 ± 15.6/54.9 ± 12.7	7 days	SFI plus CWT	CWT	Diluted in 5% GS/0.9% NS 250ml, IVGTT	NR	NR	①⑨
Yan et al. (2017)	40/40	61.68 ± 7.54/62.03 ± 7.66	21 days	SFI plus CWT	CWT	Diluted in 0.9% NS 500ml, IVGTT	d	NR	①②
You and Wang (2019)	38/38	54.67 ± 9.68/52.35 ± 10.27	7 days	SFI plus Lyophilized rhBNP	Lyophilized rhBNP	Diluted in 5% GS 250ml, IVGTT	b	NR	①⑤
Zeng (2005)	54/56	57.6 ± 15.2/56.8 ± 15.7	10 days	SFI plus CWT	CWT	Diluted in 5% GS/0.9% NS 250ml, IVGTT	k	NR	①
Zhang (2011)	37/37	54.2/55.7	14 days	SFI plus CWT	CWT	Diluted in 5% GS, IVGTT	j	Death	①
Zhang et al. (2012)	39/39	61 ± 13/61 ± 12	12 days	SFI plus CWT	CWT	Diluted in 0.9% NS 100ml, IVGTT	NR	NR	①⑪
Zhang et al. (2018)	122/122	70.47 ± 5.39/70.33 ± 5.26	14 days	SFI plus CWT	CWT	NR	NR	NR	⑨
Zhang and Zhang (2018)	38/38	63.32 ± 1.78/63.91 ± 5.86	28 days	SFI plus CWT + aspirin	CWT + aspirin	Diluted in 0.9% NS 500ml, IVGTT	NR	NR	①②⑦⑧⑨
Zhang et al. (2019)	33/32	NR	10 days	SFI plus CWT	CWT	IVGTT	c	High heart rate	②⑤⑪
Zhang (2020)	50/50	65.39 ± 3.61/65.32 ± 3.32	NR	SFI plus rhBNP	rhBNP	Diluted in 5% GS 250–500ml, IVGTT	NR	NR	①
Zhao et al. (2016)	31/31	68 ± 5/68 ± 5	7 days	SFI plus CWT + rhBNP	CWT + rhBNP	Diluted in 5% GS 250ml, IVGTT	a	Low blood pressure, arrhythmia	②③⑤⑧⑩
Zhao (2018)	60/60	63.6 ± 3.9/65.6 ± 4.1	14 days	SFI plus CWT	CWT	Diluted in 5% GS 250ml, IVGTT	NR	NR	①②⑦⑧⑨
Zong et al. (2014)	52/53	65.32 ± 12.12/65.31 ± 11.37	14 days	SFI plus CWT	CWT	Diluted in 5% GS 250ml, IVGTT	a	NR	①②⑧⑪
Zou (2013)	36/36	70 ± 4.6/70 ± 4.6	NR	SFI plus CWT	CWT	NR	NR	NR	①⑦⑧⑨

E/C: experimental group/control group; SFI: Shenfu injection; CWT: conventional western treatment; E: enoxaparin sodium; rt-PA: reverse transcriptase PA; Du: dobutamine; D: dopamine; M: metoprolol; rhBNP: recombined human; NR: not report. ①:Total effective rate; ②:LVEF: left ventricular ejection fraction; ③:LVEDD: left ventricular end-diastolic dimension; ④:SV: stroke volume; ⑤:NT-proBNP: N-terminal pro-B-type natriuretic peptide; and ⑥: adverse events; cardiac index; heart rate; cardiac output; serum creatinine; and BNP. a. WHO, diagnostic criteria; b. (Zhao, 2017); c: (Guidelines for the diagnosis and, 2015); d: (Gao, 2001); e: (Sun and Fu, 2004); f: (Luo and Lin, 2013); g: (Guidelines for the diagnosis and, 2010); h: (Randhawa et al., 2014); i: (Chen, 2008); j: (Wenwu, 2000); k: (Wang, 2002); l: (Chen, 1995); and m (Chen, 1996):

### 3.3 Risk of bias assessment

**FIGURE 2:**
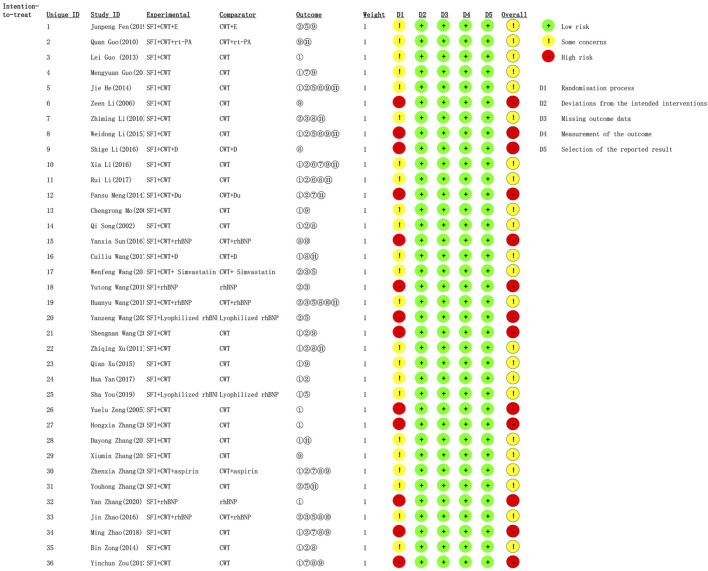
Risk of bias results for the included RCT.

All included studies published complete data and did not report selective results, so the risk of missing outcome data, measurement of the outcome, and selection of the reported result was considered as “low”. In addition, 13 (Zeng, 2005; Li et al., 2006; Zhang, 2011; Zou, 2013; Meng, 2014; Li, 2015; Li, 2016; Sun, 2016; Wang, 2018; Zhao, 2018; Wang, 2020; Zhang, 2020; Wang, 2021) articles had only one author, which led to a high risk of randomization process. In addition to these 13 studies, others studies did not state blind methods, so the risk of randomization process was considered to be some concerns. The risk of deviation was considered low because no deviation from the expected outcome was seen in any of the RCTs. Figure 2 presented the risk of bias results for the included RCTs.

### 3.4 Meta-analysis results

#### 3.4.1 Primary outcome measures of measures of NT-proBNP

**FIGURE 3:**
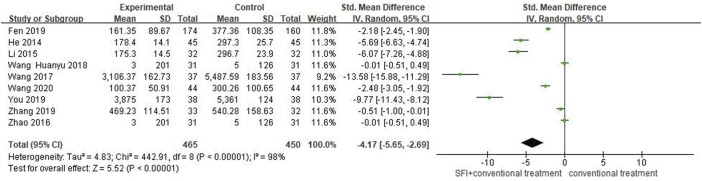
Forest plot of NT-proBNP.

Nine studies (He and Sheng, 2014; Li, 2015; Zhao et al., 2016; Wang et al., 2017; Wang et al., 2018; Fen et al., 2019; You and Wang, 2019; Zhang et al., 2019; Wang, 2020) involving 915 patients reported NT-proBNP outcomes. A random-effects model was used for meta-analysis because of high heterogeneity between studies (*p* < 0.00001, I^2^ = 98%). The sensitivity analyses did not find sources of heterogeneity. A meta regression analysis further explored that sample size, duration, age, type of disease, and usage were not the source of heterogeneity (*p* > 0.05, As Table 2 showed; for details see Supplementary Table S5). Despite lacking the source of high heterogeneity, the meta results showed that the combination of SFI and conventional medical therapy improved NT-proBNP in PAMIHF patients better than conventional medical therapy alone [SMD = −4.17; 95% CI (−5.65, −2.69); *p* < 0.00001, Figure 3]; thus, future rigorous RCTs with large sample were required to confirm this meta result.

**TABLE 2:** Meta regression analysis on the results of NT-proBNP.

_ES	Coefficient	Std. err	t	P>|t|	[95% conf. interval]	
Sample size	3.073344	6.531287	0.47	0.662	−15.06041	21.2071
Duration	−1.869973	4.332006	−0.43	0.688	−13.89755	10.1576
Usage	4.739137	6.534725	0.73	0.508	−13.40417	22.88244
Age	.8875687	6.558093	0.14	0.899	−17.32062	19.09576
_cons	−9.010748	9.910538	−0.91	0.415	−36.52681	18.50532

Sample size <50, 50–200, and ≥200 were categorized as 0, 1, and 2, respectively. Duration was categorized as 1, 2, 3, and 0, respectively, when duration<7, 7–14, ≥14 days, and no mention duration. Usage was categorized as 1, 2, 3, and 0, respectively, when it was 100 ml, 200 ml, more than 250 ml, and was no mention. Age was categorized as 0 and one when the average of participates was < 60 or ≥ 60 years old. Type of diseases was categorized as 0 and one when patients suffer from acute myocardial infarction with heart failure, accompanied without or with other disease. As for NT-proBNP, patients in the included studies suffer from acute myocardial infarction with heart failure, accompanied without or with other disease; thus, it could not be included in Meta regression analysis.

#### 3.4.2 Primary outcome measures of LVEF

**FIGURE 4:**
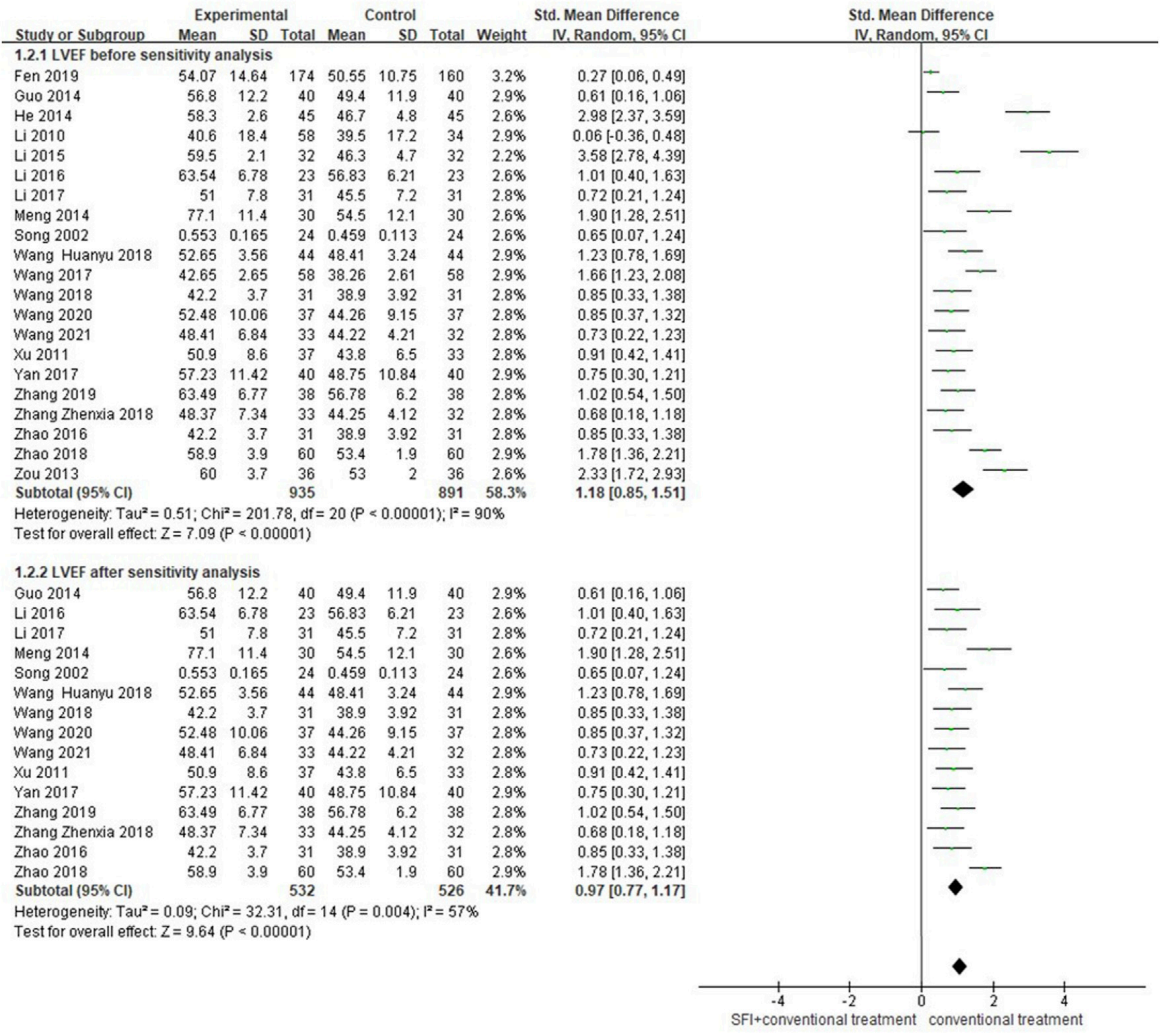
Forest plot of LVEF.

A total of 21 studies (Song et al., 2002; Li et al., 2010; Zhi-Qing et al., 2011; Zou, 2013; Guo, 2014; He and Sheng, 2014; Meng, 2014; Li, 2015; Li and Chen, 2016; Zhao et al., 2016; Li and Hou, 2017; Wang et al., 2017; Yan et al., 2017; Wang, 2018; Wang et al., 2018; Zhang and Zhang, 2018; Zhao, 2018; Fen et al., 2019; Zhang et al., 2019; Wang, 2020; Wang, 2021) involving 1826 patients reported LVEF. A random-effects model was used for meta-analysis because of high heterogeneity between studies (*p* < 0.00001, I^2^ = 90%). The results of the meta-analysis showed that the combination of SFI and conventional medical therapy improved LVEF better [RR = 1.18; 95% CI (0.85, 1.51); *p* < 0.00001, Figure 4]. The sensitivity analysis showed six studies (Zou, 2013; He and Sheng, 2014; Meng, 2014; Li, 2015; Wang et al., 2017; Fen et al., 2019) that significantly reduced the heterogeneity to 84%. Compared with other studies, two studies (He and Sheng, 2014; Li, 2015) had treatment frequency of twice a day, which may lead to high heterogeneity between studies. The meta regression analysis further explored that sample size, duration, type of diseases, age, and usage were not the main source of heterogeneity (*p* > 0.05; as shown in Table 3;for detail see Supplementary Table S5). Although, after the sensitivity analysis, the heterogeneity was still high, and the results showed that SFI combined with conventional medical therapy significantly improved LVEF in patients with PAMIHF [SMD = 0.98; 95% CI (0.71.1.24); *p* < 0.00001, Figure 4], while it required future high quality RCTs with large sample to update this meta result due to its high heterogeneity.

**TABLE 3:** Meta regression analysis on the results of LVEF.

_ES	Coefficient	Std. err	t	P>|t|	[95% conf. interval]	
Sample size	−0.270454	0.5496324	−0.49	0.630	−1.441968	0.9010597
Duration	−0.0132916	0.3310933	0.04	0.696	−0.7190003	0.6924171
Usage	−0.2900352	0.1525484	−1.90	0.077	−0.6151843	0.035114
Age	−0.239398	0.7126366	−0.34	0.742	−1.758347	1.279551
Type of disease	−1.349708	0.6562594	−2.06	0.058	−2.748492	0.0490755
_cons	2.114263	0.8478154	2.49	0.025	0.3071875	3.921339

Sample size <50, 50–200, and ≥200 were categorized as 0, 1, and 2, respectively. Duration was categorized as 1.2, 3, and 0, respectively, when duration<7, 7–14,≥14 days, and no mention duration. Usage was categorized as 1, 2, 3, and 0, respectively, when it was 100 ml, 200 ml, more than 250 ml, and was no mention. Age was categorized as 0 and one when the average of participates was < 60 or ≥ 60 years old. Type of diseases was categorized as 0 and one when patients suffer from acute myocardial infarction with heart failure, accompanied without or with other disease.

#### 3.4.3 Secondary outcome measures of total effective rate

**FIGURE 5:**
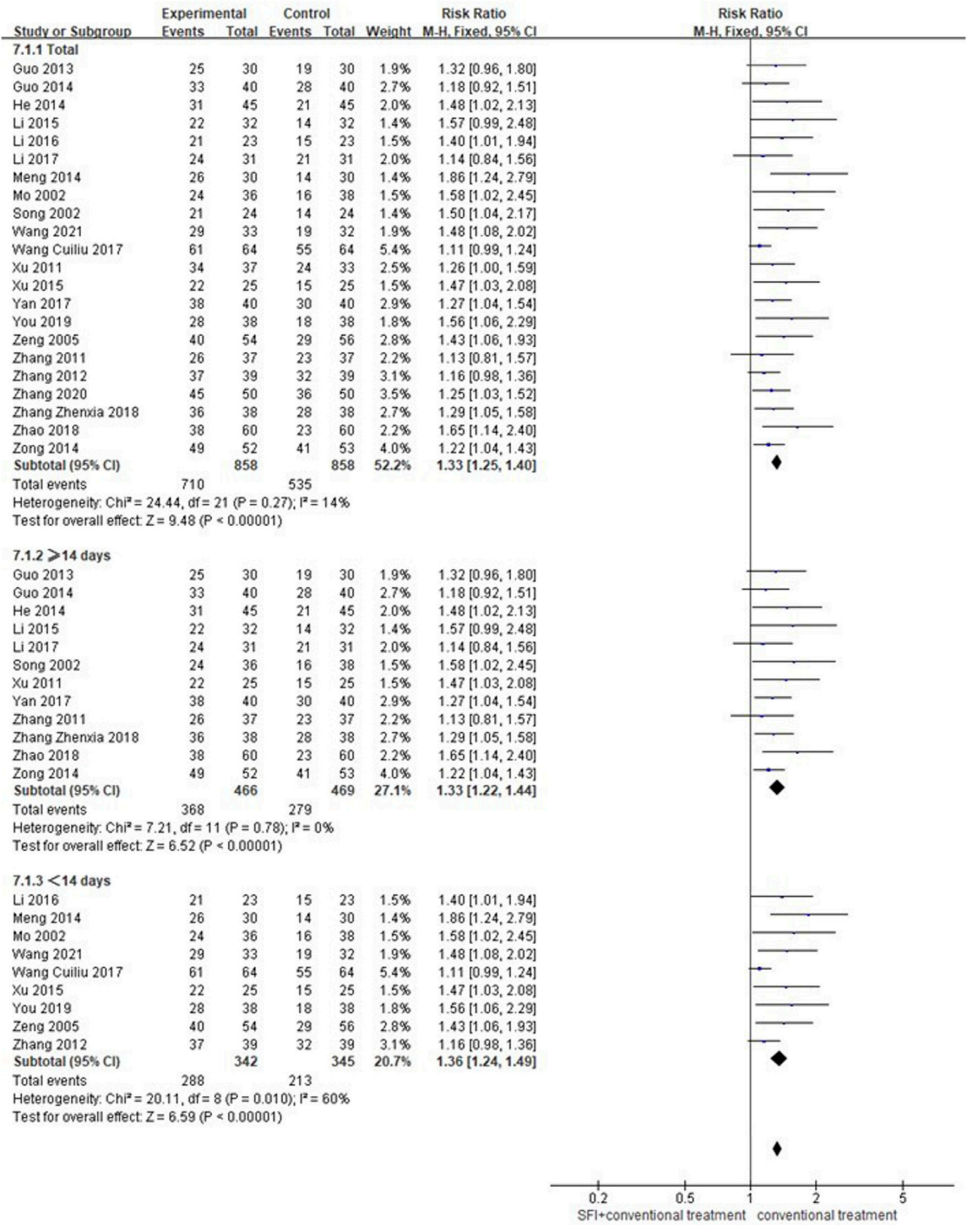
Forest plot of the total effective rate.

A total of 22 studies (Mo and Zhao, 2002; Song et al., 2002; Zeng, 2005; Zhang, 2011; Zhi-Qing et al., 2011; Zhang et al., 2012; Guo et al., 2013; Guo, 2014; He and Sheng, 2014; Meng, 2014; Zong et al., 2014; Li, 2015; Xu et al., 2015; Li and Chen, 2016; Li and Hou, 2017; Wang and Qin, 2017; Yan et al., 2017; Zhang and Zhang, 2018; Zhao, 2018; You and Wang, 2019; Zhang, 2020; Wang, 2021) involving 1716 patients reported the total effective rate. Due to low heterogeneity (*p* = 0.97, I^2^ = 0%) between-study, a fixed-effects model was used for meta-analysis. As shown in Figure 5, the results showed that the combination of SFI and conventional medication was superior to improve the total effective rate compared with conventional medication alone [RR = 1.33; 95% CI (1.25, 1.40); *p* < 0.00001]. The subgroup analysis according to the SFI dose showed < 14 days [RR = 1.33; 95% CI (1.22, 1.44); *p* < 0.00001] and ≥ 14 days [RR = 1.36; 95% CI (1.24, 1.49); *p* < 0.00001; Figure 5] both improved the total effective rate better than that of conventional medication alone.

#### 3.4.4 Secondary outcome measures of heart rate

**FIGURE 6:**
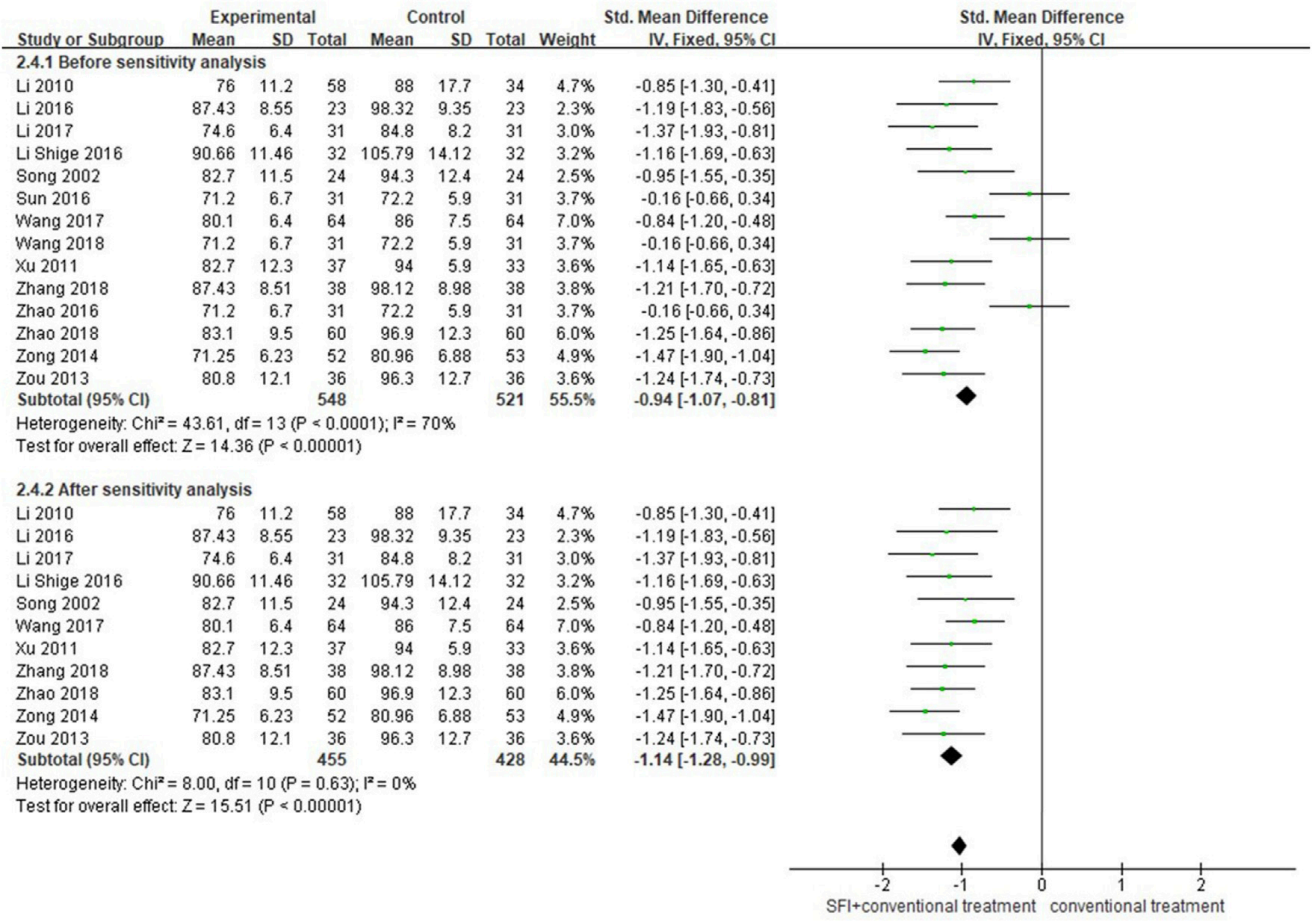
Forest plot of HR.

A total of 14 studies (Song et al., 2002; Li et al., 2010; Zhi-Qing et al., 2011; Zou, 2013; Zong et al., 2014; Li, 2016; Li and Chen, 2016; Sun, 2016; Zhao et al., 2016; Li and Hou, 2017; Wang et al., 2017; Wang et al., 2018; Zhang and Zhang, 2018; Zhao, 2018) involving 1069 patients reported the results of HR. The random-effects model was used for meta-analysis as there existed high heterogeneity between studies (*p* < 0.00001, I^2^ = 70%). After excluding three studies by using the sensitivity analysis, the heterogeneity between studies was significantly reduced to 0%. As shown in Table 1, rh-BNP plus conventional therapy was used in both the SFI group and conventional therapy groups of these three studies (Sun, 2016; Zhao et al., 2016; Wang, 2018), which may lead to heterogeneity. After the sensitivity analysis, the results showed that adjunctive use of SFI decreased the HR better than conventional medicine treatment alone [SMD = −1.14; 95% CI (−1.28, −0.99); *p* < 0.00001; Figure 6].

#### 3.4.5 Secondary outcome measures of cardiac output

**FIGURE 7:**
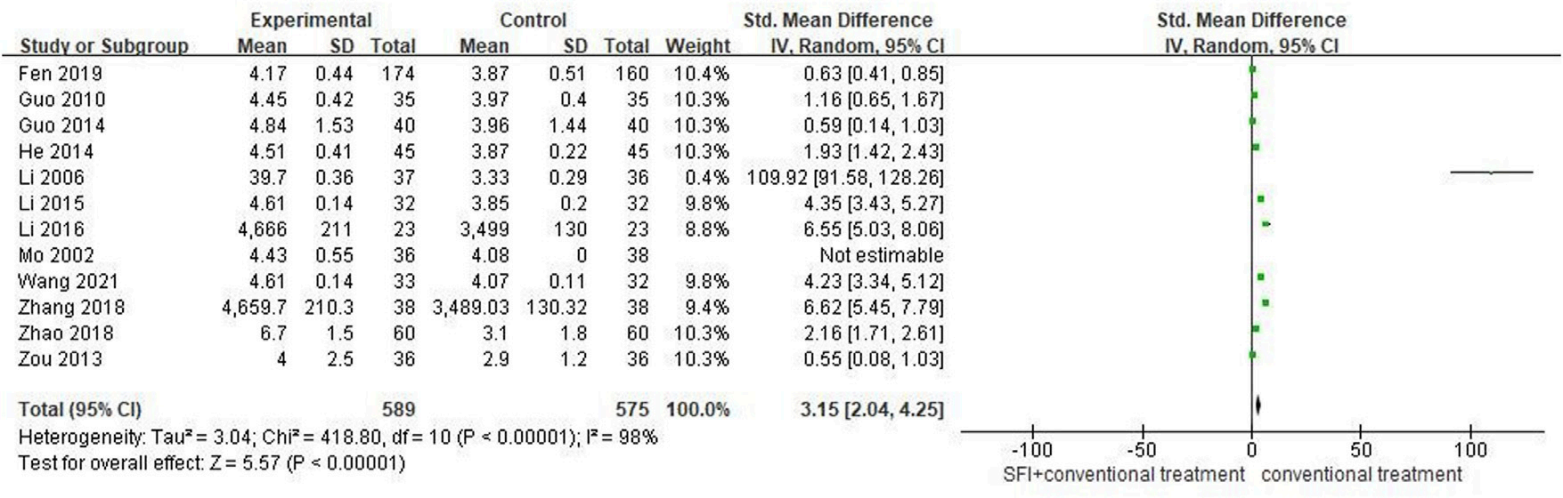
Forest plot of CO.

A total of 12 studies (Mo and Zhao, 2002; Li et al., 2006; Guo et al., 2010; Zou, 2013; Guo, 2014; He and Sheng, 2014; Li, 2015; Li and Chen, 2016; Zhang and Zhang, 2018; Zhao, 2018; Fen et al., 2019; Wang, 2021) involving 1164 patients reported the results of cardiac output (CO). The random-effects model was used for meta-analysis as there existed high heterogeneity between studies (*p* < 0.00001, I^2^ = 98%). The sensitivity analyses did not find sources of heterogeneity. The results showed that CO of PAMIHF patients was improved better by combined used of SFI and conventional medicine treatment [SMD = 3.15; 95% CI (2.04.4.25); *p* < 0.00001, Figure 7].

#### 3.4.6 Secondary outcome measures of BNP

**FIGURE 8:**
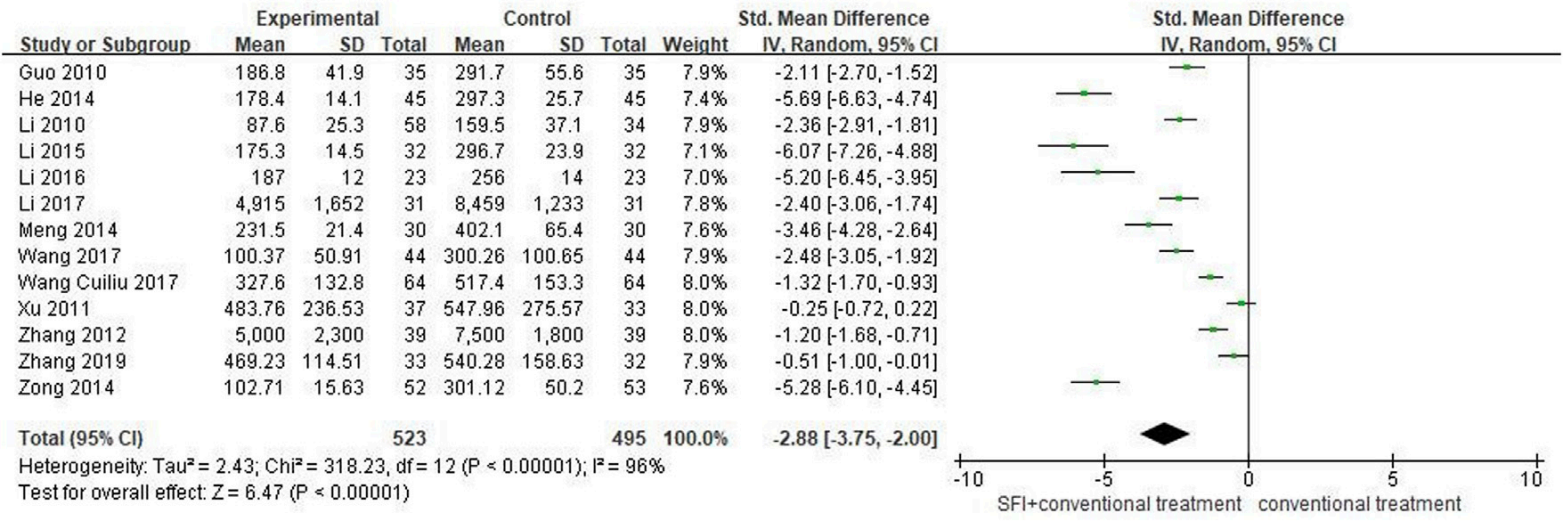
Forest plot of BNP.

A total of 13 studies (Guo et al., 2010; Li et al., 2010; Zhi-Qing et al., 2011; Zhang et al., 2012; He and Sheng, 2014; Meng, 2014; Zong et al., 2014; Li, 2015; Li and Chen, 2016; Li and Hou, 2017; Wang and Qin, 2017; Wang et al., 2017; Zhang et al., 2019) involving 1018 patients reported the value of BNP. A random-effects model was used for meta-analysis because of high heterogeneity between studies (*p* < 0.00001, I^2^ = 96%). The sensitivity analyses did not find sources of heterogeneity. The results showed that the combination of SFI and conventional medical therapy improved BNP in PAMIHF patients better than conventional medical therapy alone [SMD = −2.88; 95% CI (−3.75, −2.00); *p* < 0.00001, Figure 8].

### 3.5 Safety of adverse events comparison

**FIGURE 9:**
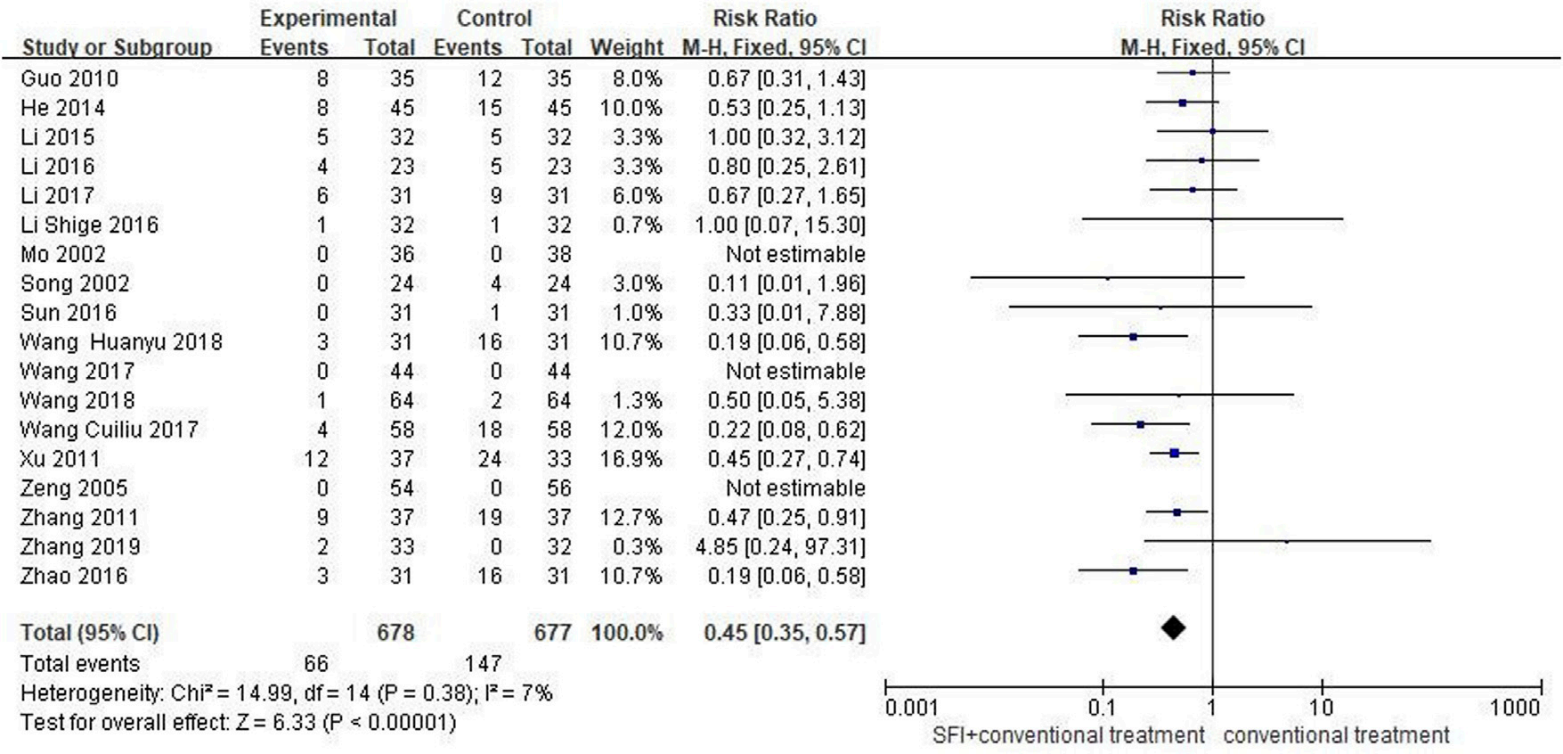
Forest plot of adverse events comparison.

A total of 18 studies (Mo and Zhao, 2002; Song et al., 2002; Zeng, 2005; Guo et al., 2010; Zhang, 2011; Zhi-Qing et al., 2011; He and Sheng, 2014; Li, 2015; Li, 2016; Li and Chen, 2016; Sun, 2016; Zhao et al., 2016; Li and Hou, 2017; Wang and Qin, 2017; Wang et al., 2017; Wang, 2018; Wang et al., 2018; Zhang et al., 2019) involving 1055 patients reported the adverse events rate. The fixed-effects model was used for meta-analysis as there existed little heterogeneity between studies (*p* = 0.38, I^2^ = 7%). The meta-analysis results showed that SFI combined with conventional medical therapy had a lower adverse event rates [RR = 0.45; 95% CI (0.35, 0.57); *p* < 0.00001, Figure 9], indicating that SFI combined with conventional treatment (9.73%, 66/678) was safer than conventional treatment alone (21.7%, 147/677).

### 3.6 Results of publication bias assess

**FIGURE 10:**
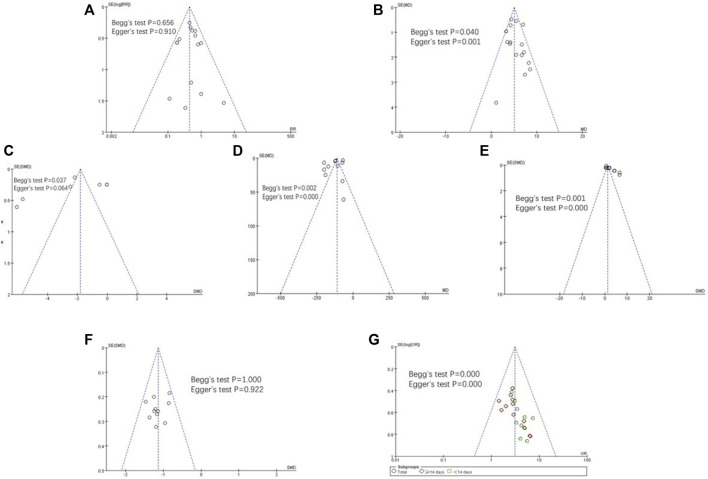
Funnel plot for publication bias assessment. **(A)** Adverse events publishes biased assessment, **(B)** LVEF publication bias assessment, **(C)** NT-proBNP publication bias assessment, **(D)** BNP publication bias assessment, **(E)** CO publishes biased assessment, **(F)** HR publishes biased assessment, and **(G)**total effective rate publication bias assessment.

We assessed publication bias for the total effective rate, LVEF, NT-proBNP, BNP, CO, HR, and adverse effect outcomes. As Figure 10 showed, the funnel plot indicted that no publication bias existed in the results of total effective rate, LVEF, HR, and adverse events as the distribution of bubbles was relatively concentrated and was not scattered on the funnel boundary. The Egger and Begg analysis suggested that no published bias existed in the results of adverse events and HR (both *p* > 0.05), while they indicated published bias existed in the results of LVEF and the total effective rate (both *p* < 0.05). However, we could not rule out the possibility of existing selective reporting of results because clinical trial registration or study protocol information was not available.

### 3.7 The quality of the evidence

We used the GRADE approach to assess the quality of evidence for the meta outcomes, which was rated from “very low” to “moderate”. They were downgraded mainly due to small sample size and unclear risk of bias for selected studies in our meta results, as shown in Table 4.

**TABLE 4:** Summary of findings by the Grading Recommendations Assessment, Development, and Evaluations (GRADE) methods.

Outcomes	No. of participants (studies)	Quality of the evidence (GRADE)	Relative effect (95%CI)	Anticipated absolute effects^*^ (95% CI)	
				Risk with [conventional medicine]	Risk with [SM injection]
Total effective rate	1716 (22 RCTs)^d^	⊕⊕⊕○	RR 3.16 (2.50–4.00)	624 per 1,000	1000 per 1,000 (1,000 to 1,000)
		Moderate^a^ risk of bias (-2^a^)			
LVEF	1564 (17 RCTs)^d^	⊕○○○	-	The mean LVEF was 0	MD 4.98 higher (4.51 higher to 5.46 higher)
		Very low^a,b^ risk of bias (-2^a^)			
		Inconsistency (-2^b^)			
NT-proBNP	219 (3 RCTs)^d^	⊕○○○	-	The mean nT-proBNP was 0	MD 119.56 lower (125.95 lower to 113.17 lower)
		Very low^a,c^			
		Risk of bias (-2^a^)			
		Inconsistency (-1^b^)			
LVEFD	328 (4 RCTs)^d^	⊕○○○	-	The mean LVEFD was 0	MD 5.84 lower (6.54 lower to 5.13 lower)
		Very low^a,c^			
		Risk of bias (-2^a^)			
		Inconsistency (-1^b^)			
BNP	1018 (13 RCTs)^d^	⊕⊕○○	-	The mean BNP was 0	MD 109.48 lower (113.66 lower to 105.29 lower)
		Low^a,b^			
		Risk of bias (-2^a^)			
		Imprecision (-1^e^)			
CI	258 (4 RCTs)^d^	⊕○○○	-	The mean CI was 0	MD 0.78 higher (0.57 higher to 0.99 higher)
		Very low^a,c^			
		Risk of bias (-2^a^)			
		Inconsistency (-1^b^)			
HR	755 (10 RCTs)^d^	⊕⊕⊕○	-	The mean heart rate was 0	MD 11.34 lower (12.75 lower to 9.93 lower)
		Moderate^a^			
		Risk of bias (-2^a^)			
CO	451 (6 RCTs)^d^	⊕⊕○○	-	The mean cardiac output was 0	MD 0.55 higher (0.5 higher to 0.61 higher)
		Low^a^			
		Risk of bias (-2^a^)			
Adverse events	1355 (18 RCTs)^d^	⊕⊕⊕○	RR 0.45 (0.35–0.57)	217 per 1,000	98 per 1,000 (76–124)
		Moderate^a^			
		Risk of bias (-2^a^)			
*The risk in the intervention group (and its 95% confidence interval) is based on the assumed risk in the comparison group and the **relative effect** of the intervention (and its 95% CI)					
**CI:** confidence interval; **MD:** mean difference; **RR:** risk ratio					
**GRADE working group grades of evidence**					
**High certainty:** we are very confident that the true effect lies close to that of the estimate of the effect					
**Moderate certainty:** we are moderately confident in the effect estimate: the true effect is likely to be close to the estimate of the effect, but there is a possibility that it is substantially different					
**Low certainty:** our confidence in the effect estimate is limited: the true effect may be substantially different from the estimate of the effect					
**Very low certainty:** we have very little confidence in the effect estimate: the true effect is likely to be substantially different from the estimate of effect					

^a^ The performance bias were high in the studies.

^b^ The direction of the effect is different as I^2^>75%.

^c^ The sample size was too small.

^d^ None of the studies stated whether there was follow-up.

## 4 Discussion

SFI has shown satisfactory clinical efficacy such as favorable neurological outcome in patients with return of spontaneous circulation after in-hospital cardiac arrest (Zhang et al., 2017; Shao et al., 2020). SFI also presents apparent effects in improving microcirculatory perfusion in patients with septic shock, and its mechanism may be related with the inhibition of endothelial dysfunction (Wang et al., 2022). Studies has shown that SFI could prevent sepsis-induced myocardial injury by inhibiting mitochondrial apoptosis (Xu et al., 2020) and attenuating lipopolysaccharide-induced myocardial inflammation (Chen et al., 2020), and it might regulate the expression of adenosine receptors to improve the myocardial ischemia–reperfusion postconditioning (Wang et al., 2021b). This systematic review and meta-analysis included 36 RCTs suggested that SFI combined with conventional western medicine had an adjunctive effect on the treatment of PAMIHF patients, which could better improve the total effective rate, LVEF, and HR. In addition, it was safer to decrease the adverse events rate compared with conventional therapy alone.

### 4.1 The adjunctive effect of SFI in treating AMI-HF

SFI has shown satisfactory clinical efficacy in the treatment of cardiovascular disease. AMI is a common acute pathological process, which can cause direct damage to the structure and function of the heart and then lead to acute HF. Because of tissue hypoperfusion and decreased coronary blood flow in PAMIHF patients, it aggravates myocardial damage, leads to increased heart rate, compensatory hypoperfusion and finally promotes myocardial remodeling (Wang, 2020). Cardiogenic shock is an extreme manifestation of PAMIHF and the leading cause of death in the AMI setting. The only treatment to reduce the mortality of patients with cardiogenic shock is early revascularization (Bahit et al., 2018). SFI could reduce the pre-load and post-load of the heart by acting on cell channels, avoiding the aggravation of myocardial hypoxia damage, promoting the repair of myocardial cells, and improving the cardiac pathology process (Wang, 2021). SFI combined with other Chinese patent medicines could inhibit the infiltration of inflammatory cells and improve hemodynamics by promoting cardiac function, reducing cardiomyocytes destruction, reducing collagen synthesis, inhibiting myocardial fibrosis, and ventricular remodeling (Gao et al., 2019). In our study, it also showed that SFI combined with conventional drug therapy improved the total effective rate, LVEF, and HR, which was consistent with previous results of published clinical studies. Interestingly, the results showed that adjunctive use of SFI showed satisfactory results regardless of treatment duration (≥14 days or <14 days) and also improved the NT-proBNP, BNP, and CO better.

### 4.2 The safety of SFI in conjunction with conventional medicine in treating AHF

In terms of clinical safety, a total of 9.7% (66/678) of adverse reactions occurred in the SFI group, while 21.7% (147/677) of adverse reactions occurred in the conventional treatment group, including nausea, vomiting, hypotension, hypertension, slow HR, and arrhythmia. With moderate safety assessment evidence, 18 studies (Mo and Zhao, 2002; Song et al., 2002; Zeng, 2005; Guo et al., 2010; Zhang, 2011; Zhi-Qing et al., 2011; He and Sheng, 2014; Li, 2015; Li, 2016; Li and Chen, 2016; Sun, 2016; Zhao et al., 2016; Li and Hou, 2017; Wang and Qin, 2017; Wang et al., 2017; Wang, 2018; Wang et al., 2018; Zhang et al., 2019) reported adverse effects, and we tentatively put forward the following arguments: combination therapy of SFI for PAMIHF was safer than conventional medicine alone. However, we still needed further eligible pivotal clinical trials to validate the safety of SFI as the risk of bias assessment of part of the RCTs was recorded as ‘unclear’.

### 4.3 The assessment of bias risk and evidence’s confidence on the meta results

We validated credible clinical evidence for our results by assessing risk of bias and confidence in the evidence. The final results indicated that detailed information on selection bias, blinding performance, and blinded outcome assessment were lacking in some of the included studies (Table 2), which may have contributed to the effect of exaggeration and reporting bias of selected outcomes. In addition, the confidence of the evidence varies from very low to moderate quality from the GRADE assessment (Table 4), and the main reasons for downgrading of evidence were risk of bias, inconsistency, imprecision, and publication bias. Thus, as the quality of the included RCTs varied, future larger RCTs with improved methodological quality were expected to further update the results of this systematic review and meta results.

### 4.4 Implications on prospective research and limitations of the present study

This study was the first systematic review and meta-analysis to summarize and evaluate the adjunctive efficacy and safety of SFI in patients with PAMIHF. Our findings suggested that SFI was safer to improve cardiac function and the total effective rate in PAMIHF. This study was designed in accordance with the high standard of methodological quality of the systematic review 2 (AMSTAR 2) by comprehensively identifying relevant literature, which improved the accuracy and clinical applicability of the systematic review.

However, there still existed limitations in this study. First, this study included 36 RCT clinical trials, most of which were small-scale clinical trials without scientific calculation before trials, and they also lacked enough follow-up time to clearly observe the long-term curative effect of SFI. Second, the quality of the part of the included studies was poor. All the studies lacked specific information about blind methods, including allocation blind, evaluation blind, or experimenter blind. Third, random grouping methods varied, few studies clearly stated that they adopted random number table method for random grouping, and most studies did not provide specific random grouping method or other methods. Fourth, the duration of treatment and the doses of SFI in the included studies were different; thus, subgroup analysis could not be performed to rule out the high heterogeneity due to unavailability of the data. In addition, due to the fact that the control group involved different conventional drug treatments, heterogeneity between studies may vary from each other. Finally, included studies in our meta-analysis were all conducted in China, which limited the generalizability of our results. Owing to the low to moderate quality of the included studies, the results should be more cautious until further rigorously trials were designed to validate the efficacy of SFI as adjuvant therapy for PAMIHF, strengthen, and update the results of the current meta-results.

In the future, the related research needs to be further improved from the following aspects: 1) the trials should be designed strictly according to the Combined Criteria for Trials Reporting (CONSORT) statements, 2) the trials should have enough follow-up time to clearly observe the long-term and short-term curative effect, 3) the sample size of the study should be large enough with scientific calculation before starting the trials, 4) there should be a clear scheme of random grouping and distribution blinding, and 5) the duration and usage of SFI should be unified to reduce the heterogeneity between studies. The curative effect and adverse reactions of SFI should be fully reported and comprehensively evaluated.

## 5 Conclusion

In conclusion, this meta-analysis suggested that SFI combined with conventional therapy was safer to significantly improve total effective rate and cardiac function in PAMIHF but due to very low to moderate quality of the meta-results evidence, which was mainly downgraded for small sample size and unclear risk of bias existed in selected studies; thus, high-quality-designed RCTs were also required for further confirmation on the efficacy and safety of adjunctive SFI therapy.

## Data Availability

The original contributions presented in the study are included in the article/Supplementary Material; further inquiries can be directed to the corresponding authors.
