# Supplementary material for: Efficacy and safety of Shenfu injection for the treatment of post-acute myocardial infarction heart failure: A systematic review and meta-analysis
Source: Front Pharmacol. 2022 Nov 24;13:1027131. doi: 10.3389/fphar.2022.1027131 (PMC9730285; doi:10.3389/fphar.2022.1027131)
Supplement: Supplementary file 3 [file Table7.DOCX]

**Details of meta regression analysis on the main outcomes**

**LVEF**

**Code:** . metareg _ES samplesize duration usage age disease, wsse(_seES) bsest(reml)

Meta-regression Number of obs = 21

REML estimate of between-study variance tau2 = 0.606

% residual variation due to heterogeneity I-squared_res = 88.14%

Proportion of between-study variance explained Adj R-squared = 6.26%

Joint test for all covariates Model F(5,15) = 1.22

With Knapp-Hartung modification Prob > F = 0.3483

| _ES | Coefficient | Std. err. | t | P>\|t\| | [95% conf. interval] | |
| --- | --- | --- | --- | --- | --- | --- |
| Sample size | -.270454 | .5496324 | -0.49 | 0.630 | -1.441968 | .9010597 |
| Duration | -.0132916 | .3310933 | 0.04 | 0.696 | -.7190003 | .6924171 |
| Usage | -.2900352 | .1525484 | -1.90 | 0.077 | -.6151843 | .035114 |
| Age | -.239398 | .7126366 | -0.34 | 0.742 | -1.758347 | 1.279551 |
| Type of disease | -1.349708 | .6562594 | -2.06 | 0.058 | -2.748492 | .0490755 |
| _cons | 2.114263 | .8478154 | 2.49 | 0.025 | .3071875 | 3.921339 |

**NT-proBNP**

**Code:** metareg _ES samplesize duration usage age, wsse(_seES) bsest(reml)

Meta-regression Number of obs = 9

REML estimate of between-study variance tau2 = 36.14

% residual variation due to heterogeneity I-squared_res = 98.90%

Proportion of between-study variance explained Adj R-squared = -65.98%

Joint test for all covariates Model F(4,4) = 0.23

With Knapp-Hartung modification Prob > F = 0.9063

| _ES | Coefficient | Std. err. | t | P>\|t\| | [95% conf. interval] | |
| --- | --- | --- | --- | --- | --- | --- |
| Sample size | 3.073344 | 6.531287 | 0.47 | 0.662 | -15.06041 | 21.2071 |
| Duration | -1.869973 | 4.332006 | -0.43 | 0.688 | -13.89755 | 10.1576 |
| Usage | 4.739137 | 6.534725 | 0.73 | 0.508 | -13.40417 | 22.88244 |
| Age | .8875687 | 6.558093 | 0.14 | 0.899 | -17.32062 | 19.09576 |
| _cons | -9.010748 | 9.910538 | -0.91 | 0.415 | -36.52681 | 18.50532 |
