# Supplementary material for: Efficacy and safety of Shenfu injection for the treatment of post-acute myocardial infarction heart failure: A systematic review and meta-analysis
Source: Front Pharmacol. 2022 Nov 24;13:1027131. doi: 10.3389/fphar.2022.1027131 (PMC9730285; doi:10.3389/fphar.2022.1027131)
Supplement: Supplementary file 4 [file Table5.DOCX]

**Supplementary file 4 The summary findings by the grading recommendations assessment, development, and evaluation (GRADE) methods.**

| **Certainty assessment** | | | | | | | **Summary of findings** | | | **Comments** |
| --- | --- | --- | --- | --- | --- | --- | --- | --- | --- | --- |
| **Participants**  **(studies)**  **Follow-up** | **Risk of bias** | **Inconsistency** | **Indirectness** | **Imprecision** | **Publication bias** | **Overall certainty of evidence** | **Events** | | **Anticipated absolute effects or Relative effect**  **(95% CI)** |  |
|  |  |  |  |  |  |  | **Control** | **Experiment** |  |  |
| Total Effective Rate  1716 (22 RCTs) | very serious^a^ | not serious | not serious | not serious | strong association | ⨁⨁⨁◯  Moderate | 710/858 (82.8%) | 535/858 (62.4%) | RR 3.16(2.50 to 4.00) | Risk of bias(-2^a^) |
| LVEF  1564 (17 RCTs) | very serious^a^ | not serious | very serious^b^ | not serious | strong association | ⨁◯◯◯  Very low | 804 | 760 | MD 4.98 higher  (4.51 higher to 5.46 higher) | Risk of bias(-2^a^)  Inconsistency(-2^b^) |
| NT-pro BNP  219 (3 RCTs) | very serious^a^ | serious^b^ | not serious | not serious | none | ⨁◯◯◯  Very low | 110 | 109 | MD 119.56 lower  (125.95 lower to 113.17 lower) | Risk of bias(-2^a^)  Inconsistency(-1^b^) |
| LVEFD  328 (4 RCTs) | very serious^a^ | serious^b^ | not serious | not serious | none | ⨁◯◯◯  Very low | 164 | 164 | MD 5.84 lower  (6.54 lower to 5.13 lower) | Risk of bias(-2^a^)  Inconsistency(-1^b^) |
| BNP  1018 (13 RCTs) | very serious^a^ | not serious | serious^c^ | not serious | strong association | ⨁⨁◯◯  Low | 525 | 495 | MD 109.48 lower  (113.66 lower to 105.29 lower) | Risk of bias(-2^a^)  Imprecision(-1^e^) |
| CI  258 (4 RCTs) | very serious^a^ | serious^b^ | not serious | not serious | none | ⨁◯◯◯  Very low | 129 | 129 | MD 0.78 higher  (0.57 higher to 0.99 higher) | Risk of bias(-2^a^)  Inconsistency(-1^b^) |
| HR  755 (10 RCTs) | very serious^a^ | not serious | not serious | not serious | strong association | ⨁⨁⨁◯  Moderate | 391 | 364 | MD 11.34 lower  (12.75 lower to 9.93 lower) | Risk of bias(-2^a^) |
| CO  451 (6 RCTs) | very serious^a^ | not serious | not serious | not serious | none | ⨁⨁◯◯  Low | 225 | 226 | MD 0.55 higher  (0.5 higher to 0.61 higher) | Risk of bias(-2^a^) |
| Adverse Events  1355 (18 RCTs) | very serious^a^ | not serious | not serious | not serious | strong association | ⨁⨁⨁◯ Moderate | 66/678 (9.7%) | 147/677 (21.7%) | RR 0.45 (0.35 to 0.57) | Risk of bias(-2^a^) |
