# Supplementary material for: Efficacy and safety of Shenfu injection for the treatment of post-acute myocardial infarction heart failure: A systematic review and meta-analysis
Source: Front Pharmacol. 2022 Nov 24;13:1027131. doi: 10.3389/fphar.2022.1027131 (PMC9730285; doi:10.3389/fphar.2022.1027131)
Supplement: Supplementary file 5 [file Table3.DOCX]

**Definition of "Total Effective Rate"**

The definition of "total effective rate" were detailed described as the following criterion for the judgment of the effect^[1]^:

Invalid rate: no change or worsening of symptoms, improvement of cardiac function <1 grade;

Effective rate: partial remission of HF, improvement of cardiac function ≥ grade 1;

Significant effective rate: HF was basically controlled, and cardiac function improved ≥2 grades.

The total effective rate=Effective rate + Significant effective rate.

Heart function level are divided according to New York Heart Association Classification ^[2]^:

NYHA Class Symptoms

I No limitation of physical activity. Ordinary physical activity does not cause undue fatigue, palpitation, dyspnea (shortness of breath).

II Slight limitation of physical activity. Comfortable at rest. Ordinary physical activity results in fatigue, palpitation, dyspnea.

III Marked limitation of physical activity. Comfortable at rest. Less than ordinary activity causes fatigue, palpitation, or dyspnea.

IV Unable to carry on any physical activity without discomfort. Symptoms of heart failure at rest. If any physical activity is undertaken, discomfort increases.

Significant effective rate includes the rate of patient's heart function improved with 2 level or more, it means that the patient’s heart function recovery from IV class to II class or even I class, from III to I class. Effective rate includes the rate of patient's heart function improved with 1 level, it means that the patient’s heart function recovery from IV class to III class, from III to II class or from II class to I class.

**参考文献**

[1] Wang S. The effect of Shenfu injection in adjuvant treatment of acute myocardial infarction complicated with heart failure. Inner Mongolia Traditional Chinese Medicine, 2021, 40(04): 127-129

[2] Zhang R, Ma S, Shanahan L, et al. Discovering and identifying New York heart association classification from electronic health records. BMC Med Inform Decis Mak, 2018, 18(Suppl 2): 48
