# Supplementary material for: Efficacy and safety of Shenfu injection for the treatment of post-acute myocardial infarction heart failure: A systematic review and meta-analysis
Source: Front Pharmacol. 2022 Nov 24;13:1027131. doi: 10.3389/fphar.2022.1027131 (PMC9730285; doi:10.3389/fphar.2022.1027131)
Supplement: Supplementary file 6 [file Table4.DOCX]

**Supplementary file S3**

Publication bias assessment results

As shown in the figure below, we assessed publication bias for other results, including Tatal effective rate, LVEF, HR, CO, NT-proBNP, BNP, and Adverse effect. All results indicated that there was no publication bias for these indicators.


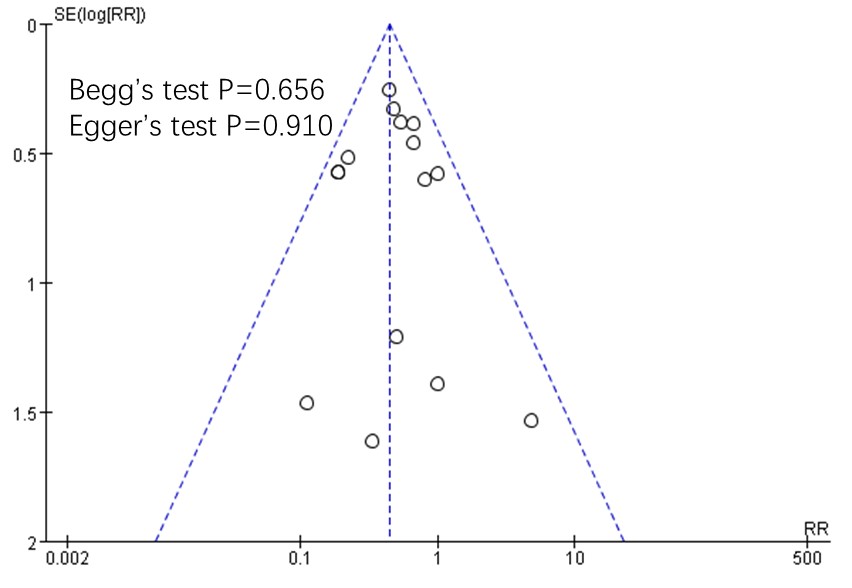


Funnel plot for publication bias assessment on Adverse events publishes biased assessment


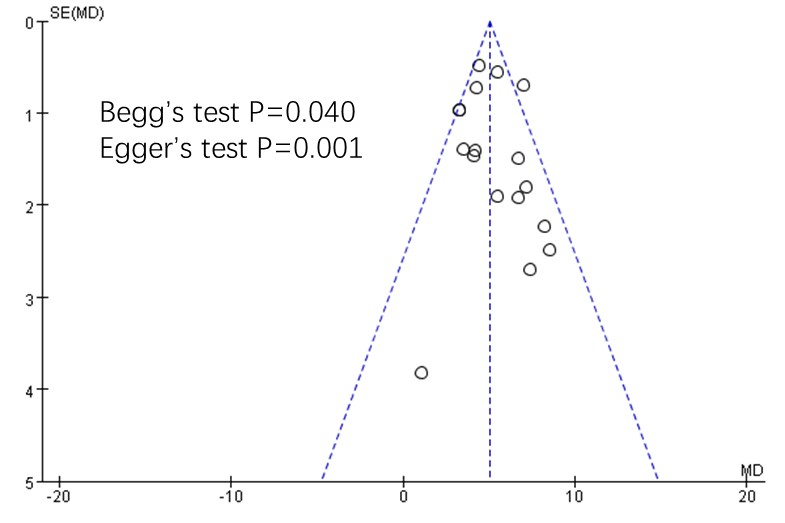


Funnel plot for publication bias assessment on LVEF


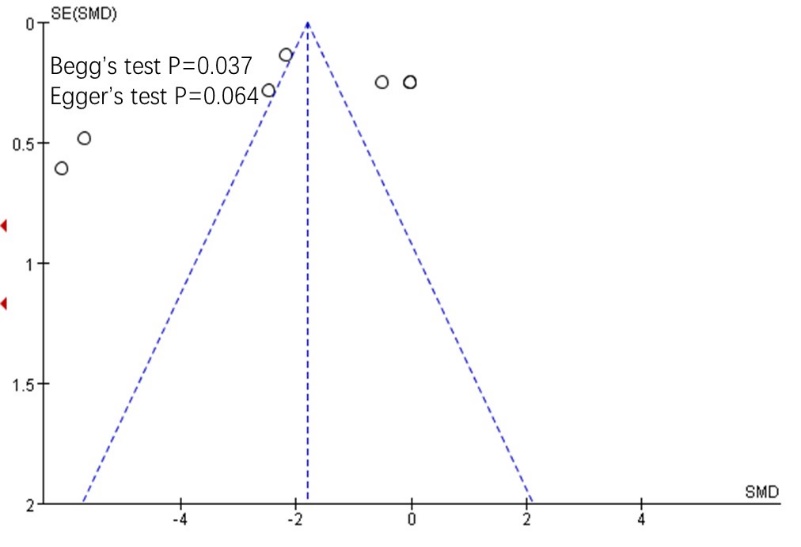


Funnel plot for publication bias assessment on NT-proBNP


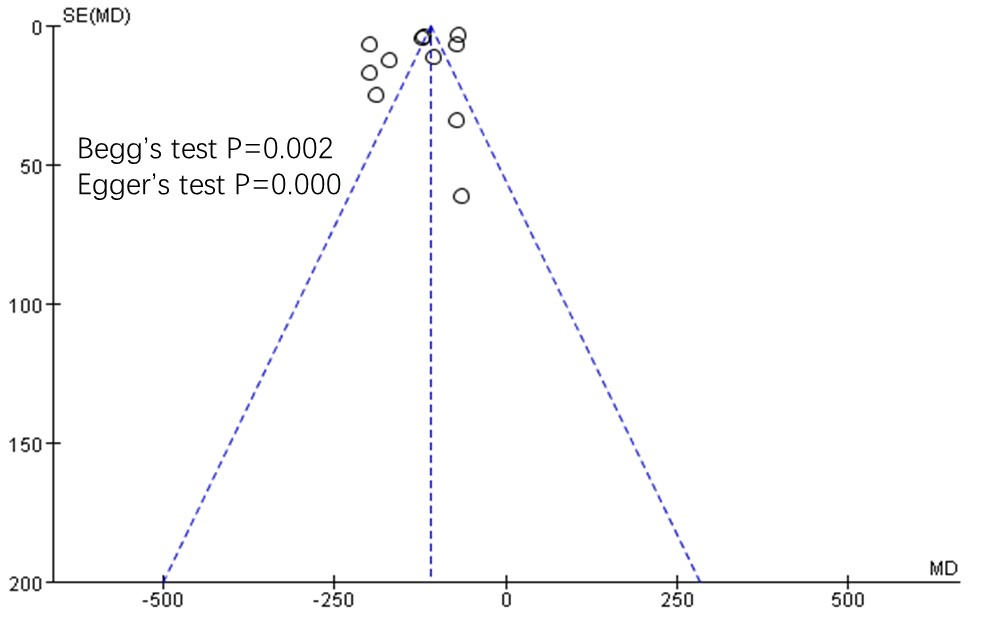


Funnel plot for publication bias assessment on BNP


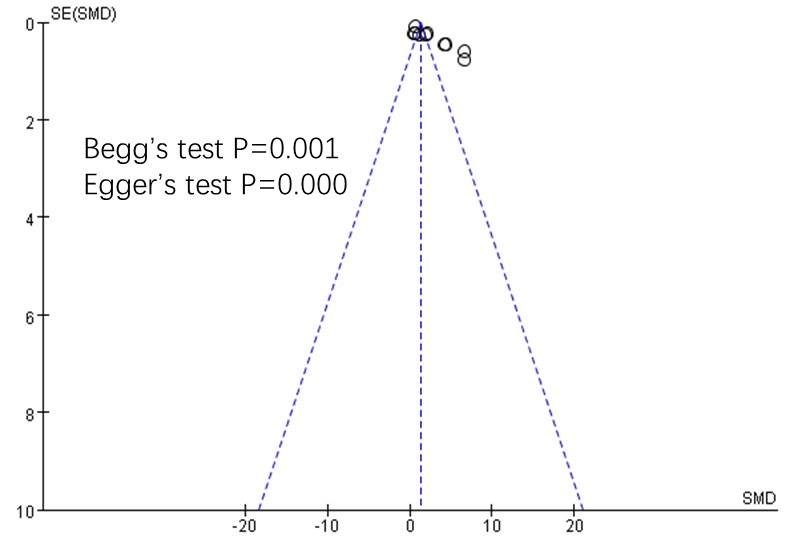


Funnel plot for publication bias assessment on CO


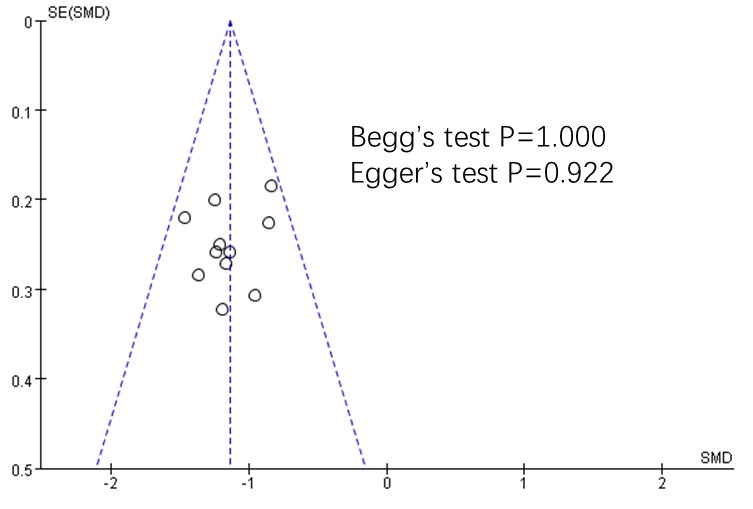


Funnel plot for publication bias assessment on HR


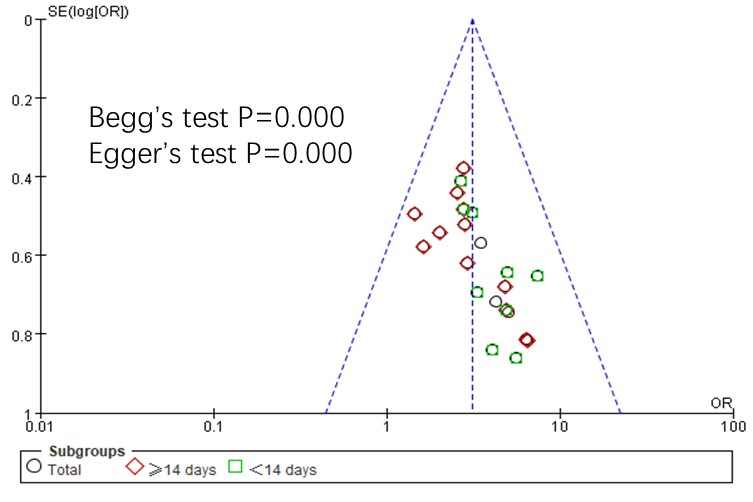


Funnel plot for publication bias assessment on Total effective rate

In addition, we also used egg and begg to detect publication bias, as shown in the figure below.

Table2 Egger and begg test for published bias of included studies

| Tatal effective rate | Egger test | | Begg test | |
| --- | --- | --- | --- | --- |
| Std_Eff | T | P | Z | P |
| Experimental | -0.18 | 0.858 | 3.98 | 0.000 |
| control | 6.90 | 0.000 | 3.95 | 0.000 |

| LVEF | Egger test | | Begg test | |
| --- | --- | --- | --- | --- |
| Std_Eff | T | P | Z | P |
| Experimental | -1.42 | 0.171 | 2.08 | 0.037 |
| control | 3.77 | 0.001 | 2.05 | 0.040 |

| HR | Egger test | | Begg test | |
| --- | --- | --- | --- | --- |
| Std_Eff | T | P | Z | P |
| Experimental | -1.22 | 0.245 | 0.00 | 1.000 |
| control | 0.10 | 0.922 | -0.05 | 1.000 |

| NT-proBNP | Egger test | | Begg test | |
| --- | --- | --- | --- | --- |
| Std_Eff | T | P | Z | P |
| Experimental | 0.26 | 0.806 | -2.19 | 0.029 |
| control | -2.20 | 0.064 | 2.09 | 0.037 |

| BNP | Egger test | | Begg test | |
| --- | --- | --- | --- | --- |
| Std_Eff | T | P | Z | P |
| Experimental | 2.90 | 0.014 | -3.17 | 0.002 |
| control | -5.94 | 0.000 | 3.11 | 0.002 |

| Cardiac output | Egger test | | Begg test | |
| --- | --- | --- | --- | --- |
| Std_Eff | T | P | Z | P |
| Experimental | -2.29 | 0.048 | 3.50 | 0.000 |
| control | 6.88 | 0.000 | 3.43 | 0.001 |

| Adverse effect | Egger test | | Begg test | |
| --- | --- | --- | --- | --- |
| Std_Eff | T | P | Z | P |
| Experimental | -2.70 | 0.018 | 0.49 | 0.621 |
| control | 0.12 | 0.910 | 0.45 | 0.656 |
