# Supplementary material for: Efficacy and safety of Shenfu injection for the treatment of post-acute myocardial infarction heart failure: A systematic review and meta-analysis
Source: Front Pharmacol. 2022 Nov 24;13:1027131. doi: 10.3389/fphar.2022.1027131 (PMC9730285; doi:10.3389/fphar.2022.1027131)
Supplement: Supplementary file 7 [file Table6.DOCX]

**Search strategy and search results for the bibliographic databases.**

1. Pubmed database

| Iterm | MeSH | Study number |
| --- | --- | --- |
| #1 | ("shenfu injection"[Title/Abstract] OR "SFI"[Title/Abstract]) AND (fft[Filter]) | 1027 |
| #2 | ("acute myocardial infarction"[Title/Abstract] OR "diaphragmatic myocardial infarction"[Title/Abstract] OR (("diaphragm"[MeSH Terms] OR "diaphragm"[All Fields] OR "Diaphragmatic"[All Fields]) AND "myocardial infarctions"[Title/Abstract]) OR (("infarctation"[All Fields] OR "infarcted"[All Fields] OR "infarctic"[All Fields] OR "infarcting"[All Fields] OR "Infarction"[MeSH Terms] OR "Infarction"[All Fields] OR "infarct"[All Fields] OR "Infarctions"[All Fields] OR "infarcts"[All Fields] OR "infarctive"[All Fields]) AND "diaphragmatic myocardial"[Title/Abstract]) OR (("infarctation"[All Fields] OR "infarcted"[All Fields] OR "infarctic"[All Fields] OR "infarcting"[All Fields] OR "Infarction"[MeSH Terms] OR "Infarction"[All Fields] OR "infarct"[All Fields] OR "Infarctions"[All Fields] OR "infarcts"[All Fields] OR "infarctive"[All Fields]) AND "diaphragmatic myocardial"[Title/Abstract]) OR (("myocardial infarction"[MeSH Terms] OR ("Myocardial"[All Fields] AND "Infarction"[All Fields]) OR "myocardial infarction"[All Fields]) AND "Diaphragmatic"[Title/Abstract]) OR (("myocardial infarction"[MeSH Terms] OR ("Myocardial"[All Fields] AND "Infarction"[All Fields]) OR "myocardial infarction"[All Fields] OR ("Myocardial"[All Fields] AND "Infarctions"[All Fields]) OR "myocardial infarctions"[All Fields]) AND "Diaphragmatic"[Title/Abstract]) OR "myocardial infarction inferior wall"[Title/Abstract] OR "inferior myocardial infarction"[Title/Abstract] OR "infarction inferior myocardial"[Title/Abstract] OR (("infarctation"[All Fields] OR "infarcted"[All Fields] OR "infarctic"[All Fields] OR "infarcting"[All Fields] OR "Infarction"[MeSH Terms] OR "Infarction"[All Fields] OR "infarct"[All Fields] OR "Infarctions"[All Fields] OR "infarcts"[All Fields] OR "infarctive"[All Fields]) AND "inferior myocardial"[Title/Abstract]) OR "inferior myocardial infarctions"[Title/Abstract] OR "myocardial infarction inferior"[Title/Abstract] OR (("myocardial infarction"[MeSH Terms] OR ("Myocardial"[All Fields] AND "Infarction"[All Fields]) OR "myocardial infarction"[All Fields] OR ("Myocardial"[All Fields] AND "Infarctions"[All Fields]) OR "myocardial infarctions"[All Fields]) AND "Inferior"[Title/Abstract]) OR "acute inferior myocardial infarction"[Title/Abstract] OR "myocardial infarction anterior wall"[Title/Abstract] OR "anterolateral myocardial infarction"[Title/Abstract] OR "anterolateral myocardial infarctions"[Title/Abstract] OR (("infarctation"[All Fields] OR "infarcted"[All Fields] OR "infarctic"[All Fields] OR "infarcting"[All Fields] OR "Infarction"[MeSH Terms] OR "Infarction"[All Fields] OR "infarct"[All Fields] OR "Infarctions"[All Fields] OR "infarcts"[All Fields] OR "infarctive"[All Fields]) AND "anterolateral myocardial"[Title/Abstract]) OR (("infarctation"[All Fields] OR "infarcted"[All Fields] OR "infarctic"[All Fields] OR "infarcting"[All Fields] OR "Infarction"[MeSH Terms] OR "Infarction"[All Fields] OR "infarct"[All Fields] OR "Infarctions"[All Fields] OR "infarcts"[All Fields] OR "infarctive"[All Fields]) AND "anterolateral myocardial"[Title/Abstract]) OR "myocardial infarction anterolateral"[Title/Abstract] OR (("myocardial infarction"[MeSH Terms] OR ("Myocardial"[All Fields] AND "Infarction"[All Fields]) OR "myocardial infarction"[All Fields] OR ("Myocardial"[All Fields] AND "Infarctions"[All Fields]) OR "myocardial infarctions"[All Fields]) AND "Anterolateral"[Title/Abstract]) OR "anteroseptal myocardial infarction"[Title/Abstract] OR "anteroseptal myocardial infarctions"[Title/Abstract] OR (("infarctation"[All Fields] OR "infarcted"[All Fields] OR "infarctic"[All Fields] OR "infarcting"[All Fields] OR "Infarction"[MeSH Terms] OR "Infarction"[All Fields] OR "infarct"[All Fields] OR "Infarctions"[All Fields] OR "infarcts"[All Fields] OR "infarctive"[All Fields]) AND "anteroseptal myocardial"[Title/Abstract]) OR (("infarctation"[All Fields] OR "infarcted"[All Fields] OR "infarctic"[All Fields] OR "infarcting"[All Fields] OR "Infarction"[MeSH Terms] OR "Infarction"[All Fields] OR "infarct"[All Fields] OR "Infarctions"[All Fields] OR "infarcts"[All Fields] OR "infarctive"[All Fields]) AND "anteroseptal myocardial"[Title/Abstract]) OR "myocardial infarction anteroseptal"[Title/Abstract] OR (("myocardial infarction"[MeSH Terms] OR ("Myocardial"[All Fields] AND "Infarction"[All Fields]) OR "myocardial infarction"[All Fields] OR ("Myocardial"[All Fields] AND "Infarctions"[All Fields]) OR "myocardial infarctions"[All Fields]) AND "Anteroseptal"[Title/Abstract]) OR "acute anterior wall myocardial infarction"[Title/Abstract]) AND (fft[Filter]) | 54457 |
| #3 | ("heart failure"[Title/Abstract] OR "cardiac failure"[Title/Abstract] OR "myocardial failure"[Title/Abstract] OR "heart failure left sided"[Title/Abstract] OR "heart failure left sided"[Title/Abstract] OR "left sided heart failure"[Title/Abstract] OR "left sided heart failure"[Title/Abstract] OR "heart failure right sided"[Title/Abstract] OR "heart failure right sided"[Title/Abstract] OR "right sided heart failure"[Title/Abstract] OR "right sided heart failure"[Title/Abstract] OR "congestive heart failure"[Title/Abstract] OR "heart failure congestive"[Title/Abstract] OR "heart decompensation"[Title/Abstract] OR "decompensation heart"[Title/Abstract] OR "acute heart failure"[Title/Abstract]) AND (fft[Filter]) | 184575 |
| #4 | ("conventional treatment"[Title/Abstract] OR "therapies complementary"[Title/Abstract] OR "therapy complementary"[Title/Abstract] OR "complementary medicine"[Title/Abstract] OR "medicine complementary"[Title/Abstract] OR "alternative medicine"[Title/Abstract] OR "medicine alternative"[Title/Abstract] OR "alternative therapies"[Title/Abstract] OR "therapies alternative"[Title/Abstract] OR "therapy alternative"[Title/Abstract]) AND (fft[Filter]) | 28196 |
| #5 | ("randomised controlled trial"[Title/Abstract] OR "clinical trials randomized"[Title/Abstract] OR "trials randomized clinical"[Title/Abstract] OR "controlled clinical trials randomized"[Title/Abstract] OR "veterinary randomized controlled trial"[Title/Abstract] OR "non inferiority trial"[Title/Abstract] OR "noninferiority trial"[Title/Abstract] OR "superiority trial"[Title/Abstract] OR "equivalence clinical trial"[Title/Abstract]) AND (fft[Filter]) | 32089 |
| #6 | #1 AND #2 AND #3 AND #4 AND #5 | 0 |

1. Embase database

| Iterm | MeSH | Study number |
| --- | --- | --- |
| #1 | 'shenfu injection'/exp OR 'shenfu injection' OR (('shenfu'/exp OR shenfu) AND ('injection'/exp OR injection)) OR sfi | 2700 |
| #2 | 'diaphragmatic myocardial infarction' OR (diaphragmatic myocardial AND ('infarction'/exp OR infarction)) OR 'diaphragmatic myocardial infarctions' OR (diaphragmatic AND myocardial AND infarctions) OR 'infarction, diaphragmatic myocardial' OR (('infarction,'/exp OR infarction,) AND diaphragmatic AND myocardial) OR 'infarctions, diaphragmatic myocardial' OR (infarctions, AND diaphragmatic AND myocardial) OR 'myocardial infarction, diaphragmatic' OR (myocardial AND ('infarction,'/exp OR infarction,) AND diaphragmatic) OR 'myocardial infarctions, diaphragmatic' OR (myocardial AND infarctions, AND diaphragmatic) OR 'myocardial infarction, inferior wall' OR (myocardial AND ('infarction,'/exp OR infarction,) AND inferior AND wall) OR 'inferior myocardial infarction' OR (inferior myocardial AND ('infarction'/exp OR infarction)) OR 'infarction, inferior myocardial' OR (('infarction,'/exp OR infarction,) AND inferior AND myocardial) OR 'infarctions, inferior myocardial' OR (infarctions, AND inferior AND myocardial) OR 'inferior myocardial infarctions' OR (inferior AND myocardial AND infarctions) OR 'myocardial infarction, inferior' OR (myocardial AND ('infarction,'/exp OR infarction,) AND inferior) OR 'myocardial infarctions, inferior' OR (myocardial AND infarctions, AND inferior) OR 'acute inferior myocardial infarction' OR (acute inferior myocardial AND ('infarction'/exp OR infarction)) OR 'myocardial infarction, anterior wall' OR (myocardial AND ('infarction,'/exp OR infarction,) AND anterior AND wall) OR 'anterolateral myocardial infarction' OR (anterolateral myocardial AND ('infarction'/exp OR infarction)) OR 'anterolateral myocardial infarctions' OR (anterolateral AND myocardial AND infarctions) OR 'infarction, anterolateral myocardial' OR (('infarction,'/exp OR infarction,) AND anterolateral AND myocardial) OR 'infarctions, anterolateral myocardial ormyocardial infarction, anterolateral' OR (infarctions, AND myocardial AND ormyocardial AND ('infarction,'/exp OR infarction,) AND anterolateral) OR 'myocardial infarctions, anterolateral' OR (myocardial AND infarctions, AND anterolateral) OR 'anteroseptal myocardial infarction' OR (anteroseptal myocardial AND ('infarction'/exp OR infarction)) OR 'anteroseptal myocardial infarctions' OR (anteroseptal AND myocardial AND infarctions) OR 'infarction, anteroseptal myocardial' OR (('infarction,'/exp OR infarction,) AND anteroseptal AND myocardial) OR 'infarctions, anteroseptal myocardial' OR (infarctions, AND anteroseptal AND myocardial) OR 'myocardial infarction, anteroseptal' OR (myocardial AND ('infarction,'/exp OR infarction,) AND anteroseptal) OR 'myocardial infarctions, anteroseptal' OR (myocardial AND infarctions, AND anteroseptal) OR 'acute anterior wall myocardial infarction' OR (acute anterior AND wall myocardial AND ('infarction'/exp OR infarction)) | 17976 |
| #3 | 'heart failure'/exp OR 'heart failure' OR (('heart'/exp OR heart) AND ('failure'/exp OR failure)) OR 'acute heart failure'/exp OR 'acute heart failure' OR (acute AND ('heart'/exp OR heart) AND ('failure'/exp OR failure)) OR 'cardiac failure'/exp OR 'cardiac failure' OR (('cardiac'/exp OR cardiac) AND ('failure'/exp OR failure)) OR 'myocardial failure'/exp OR 'myocardial failure' OR (myocardial AND ('failure'/exp OR failure)) OR 'heart failure, left-sided orheart failure, left sided' OR (('heart'/exp OR heart) AND 'left sided' AND orheart AND failure, AND left AND sided) OR 'left-sided heart failure' OR ('left sided heart' AND ('failure'/exp OR failure)) OR 'left sided heart failure' OR (left AND sided heart AND ('failure'/exp OR failure)) OR 'heart failure, right-sided' OR (('heart'/exp OR heart) AND failure, AND 'right sided') OR 'heart failure, right sided' OR (('heart'/exp OR heart) AND failure, AND right AND sided) OR 'right-sided heart failure' OR ('right sided heart' AND ('failure'/exp OR failure)) OR 'right sided heart failure' OR (right AND sided heart AND ('failure'/exp OR failure)) OR 'congestive heart failure' OR (congestive heart AND ('failure'/exp OR failure)) OR 'heart failure, congestive'/exp OR 'heart failure, congestive' OR (('heart'/exp OR heart) AND failure, AND congestive) OR 'heart decompensation'/exp OR 'heart decompensation' OR (('heart'/exp OR heart) AND decompensation) OR 'decompensation, heart'/exp OR 'decompensation, heart' OR (decompensation, AND ('heart'/exp OR heart)) | 844284 |
| #4 | 'randomised controlled trial'/exp OR 'randomised controlled trial' OR (randomised AND controlled AND ('trial'/exp OR trial)) OR 'clinical trials, randomized' OR (('clinical'/exp OR clinical) AND trials, AND randomized) OR 'trials, randomized clinical' OR (trials, AND randomized AND ('clinical'/exp OR clinical)) OR 'controlled clinical trials, randomized' OR (controlled AND ('clinical'/exp OR clinical) AND trials, AND randomized) OR 'veterinary randomized controlled trial' OR (veterinary randomized AND controlled AND ('trial'/exp OR trial)) OR 'non-inferiority trial'/exp OR 'non-inferiority trial' OR ('non inferiority' AND ('trial'/exp OR trial)) OR 'noninferiority trial'/exp OR 'noninferiority trial' OR (noninferiority AND ('trial'/exp OR trial)) OR 'superiority trial'/exp OR 'superiority trial' OR (superiority AND ('trial'/exp OR trial)) OR 'equivalence clinical trial'/exp OR 'equivalence clinical trial' OR (equivalence AND ('clinical'/exp OR clinical) AND ('trial'/exp OR trial)) | 964054 |
| #5 | 'conventional treatment' OR (conventional AND ('treatment'/exp OR treatment)) OR 'therapies, complementary' OR (therapies, AND complementary) OR 'therapy, complementary' OR (('therapy,'/exp OR therapy,) AND complementary) OR 'complementary medicine'/exp OR 'complementary medicine' OR (complementary AND ('medicine'/exp OR medicine)) OR 'medicine, complementary' OR (('medicine,'/exp OR medicine,) AND complementary) OR 'alternative medicine'/exp OR 'alternative medicine' OR (alternative AND ('medicine'/exp OR medicine)) OR 'medicine, alternative' OR (('medicine,'/exp OR medicine,) AND alternative) OR 'alternative therapies'/exp OR 'alternative therapies' OR (alternative AND therapies) OR 'therapies, alternative' OR (therapies, AND alternative) OR 'therapy, alternative' OR (('therapy,'/exp OR therapy,) AND alternative) | 959217 |
| #6 | #1 AND #2 AND #3 AND #4 AND #5 | 0 |

1. Web of Science

| Iterm | MeSH | Study number |
| --- | --- | --- |
| #1 | (TS=(Shenfu Injection)) OR TS=(SFI) | 2167 |
| #2 | (((((((((((((((((((((((((((TS=(Diaphragmatic Myocardial Infarction)) OR TS=(Diaphragmatic Myocardial Infarctions)) OR TS=(Infarction, Diaphragmatic Myocardial)) OR TS=(Infarctions, Diaphragmatic Myocardial)) OR TS=(Myocardial Infarction, Diaphragmatic)) OR TS=(Myocardial Infarctions, Diaphragmatic)) OR TS=(Myocardial Infarction, Inferior Wall)) OR TS=(Inferior Myocardial Infarction)) OR TS=(Infarction, Inferior Myocardial)) OR TS=(Infarctions, Inferior Myocardial)) OR TS=(Inferior Myocardial Infarctions)) OR TS=(Myocardial Infarction, Inferior)) OR TS=(Myocardial Infarctions, Inferior)) OR TS=(Acute Inferior Myocardial Infarction)) OR TS=(Myocardial Infarction, Anterior Wall)) OR TS=(Anterolateral Myocardial Infarction)) OR TS=(Anterolateral Myocardial Infarctions)) OR TS=(Infarction, Anterolateral Myocardial)) OR TS=(Infarctions, Anterolateral Myocardial)) OR TS=(Myocardial Infarction, Anterolateral)) OR TS=(Myocardial Infarctions, Anterolateral)) OR TS=(Anteroseptal Myocardial Infarction)) OR TS=(Anteroseptal Myocardial Infarctions)) OR TS=(Infarction, Anteroseptal Myocardial)) OR TS=(Infarctions, Anteroseptal Myocardial)) OR TS=(Myocardial Infarction, Anteroseptal)) OR TS=(Myocardial Infarctions, Anteroseptal)) OR TS=(Acute Anterior Wall Myocardial Infarction) | 14982 |
| #3 | (((((((((((((((TS=(Heart Failure)) OR TS=(Acute heart failure)) OR TS=(Cardiac Failure)) OR TS=(Myocardial Failure)) OR TS=(Heart Failure, Left-Sided)) OR TS=(Heart Failure, Left Sided)) OR TS=(Left-Sided Heart Failure)) OR TS=(Left Sided Heart Failure)) OR TS=(Heart Failure, Right-Sided)) OR TS=(Heart Failure, Right Sided)) OR TS=(Right-Sided Heart Failure)) OR TS=(Right Sided Heart Failure)) OR TS=(Congestive Heart Failure)) OR TS=(Heart Failure, Congestive)) OR TS=(Heart Decompensation)) OR TS=(Decompensation, Heart) | 543581 |
| #4 | ((((((((TS=(randomised controlled trial)) OR TS=(Clinical Trials, Randomized)) OR TS=(Trials, Randomized Clinical)) OR TS=(Controlled Clinical Trials, Randomized)) OR TS=(Veterinary Randomized Controlled Trial)) OR TS=(Non-Inferiority Trial)) OR TS=(Noninferiority Trial)) OR TS=(Superiority Trial)) OR TS=(Equivalence Clinical Trial) | 826610 |
| #5 | (((((((((TS=(conventional treatment)) OR TS=(Therapies, Complementary)) OR TS=(Therapy, Complementary)) OR TS=(Complementary Medicine)) OR TS=(Medicine, Complementary)) OR TS=(Alternative Medicine)) OR TS=(Medicine, Alternative)) OR TS=(Alternative Therapies)) OR TS=(Therapies, Alternative)) OR TS=(Therapy, Alternative) | 722078 |
| #6 | #1 AND #2 AND #3 AND #4 AND #5 | 1 |

1. China National Knowledge Infrastructure (CNKI)) database (In Chinese)

| Iterm | MeSH | Study number |
| --- | --- | --- |
| #1 | (主题=参附注射液) OR (篇名=参附注射液) OR (篇关摘=参附注射液) | 4445 |
| #2 | (主题=急性心肌梗死) OR (篇名=急性心肌梗死) OR (篇关摘=急性心肌梗死) OR (主题=急性心肌梗塞) OR (篇名=急性心肌梗塞) OR (篇关摘=急性心肌梗塞) | 86312 |
| #3 | (主题=心力衰竭) OR (篇名=心力衰竭) OR (篇关摘=心力衰竭) OR (主题=急性心力衰竭) OR (篇名=急性心力衰竭) OR (篇关摘=急性心力衰竭) OR (主题=慢性心力衰竭) OR (篇名=慢性心力衰竭) OR (篇关摘=慢性心力衰竭) | 132879 |
| #4 | (主题=随机对照) OR (篇名=随机对照) OR (篇关摘=随机对照) | 48250 |
| #5 | (主题=西药治疗) OR (篇名=西药治疗) OR (篇关摘=西药治疗) OR (主题=常规治疗) OR (篇名=常规治疗) OR (篇关摘=常规治疗) OR (主题=药物治疗) OR (篇名=药物治疗) OR (篇关摘=药物治疗) | 151675 |
| #6 | #1 AND #2 AND #3 AND #4 AND #5 | 45 |

1. Chinese Biomedical Literature Database (CBM) database (In Chinese)

| Iterm | MeSH | Study number |
| --- | --- | --- |
| #1 | "参附注射液"[标题:智能] OR "参附注射液"[摘要:智能] | 3453 |
| #2 | "急性心肌梗死"[标题:智能] OR "急性心肌梗死"[摘要] OR "急性心肌梗塞"[标题:智能] OR "急性心肌梗塞"[摘要:智能] | 65219 |
| #3 | "心力衰竭"[常用字段:智能] OR "心力衰竭"[常用字段:智能] OR "急性心力衰竭"[常用字段:智能] OR "急性心力衰竭"[常用字段:智能] OR "慢性心力衰竭"[常用字段:智能] OR "慢性心力衰竭"[常用字段:智能] | 125882 |
| #4 | "随机对照"[标题:智能] OR "随机对照"[摘要:智能] | 228676 |
| #5 | "西药治疗"[摘要:智能] OR "西药治疗"[标题:智能] OR "常规治疗"[标题:智能] OR "常规治疗"[摘要:智能] OR "药物治疗"[标题:智能] OR "药物治疗"[摘要:智能] | 940287 |
| #6 | #1 AND #2 AND #3 AND #4 AND #5 | 57 |

1. Wan-fang Database (In Chinese)

| Iterm | MeSH | Study number |
| --- | --- | --- |
| #1 | 题名或关键词:("参附注射液") + 主题:("参附注射液") + 摘要:("参附注射液") | 352 |
| #2 | 题名或关键词:("急性心肌梗死") + 主题:("急性心肌梗死") + 摘要:("急性心肌梗死") + 题名或关键词:("急性心肌梗塞") + 主题:("急性心肌梗塞") + 摘要:("急性心肌梗塞") | 110692 |
| #3 | 题名或关键词:("心力衰竭") + 主题:("心力衰竭") + 摘要:("心力衰竭") + 题名或关键词:("急性心力衰竭") + 主题:("急性心力衰竭") + 摘要:("急性心力衰竭")+ 题名或关键词:("慢性心力衰竭") + 主题:("慢性心力衰竭") + 摘要:("慢性心力衰竭") | 67315 |
| #4 | 题名或关键词:("随机对照") + 主题:("随机对照") + 摘要:("随机对照") | 2640 |
| #5 | 题名或关键词:("西药治疗") + 主题:("西药治疗") + 摘要:("西药治疗") + 题名或关键词:("常规治疗") + 主题:("常规治疗") + 摘要:("常规治疗") + 题名或关键词:("药物治疗") + 主题:(" 药物治疗") + 摘要:(" 药物治疗") | 36544 |
| #6 | #1 AND #2 AND #3 AND #4 AND #5 | 60 |

1. China Science and Technology Journal Database (VIP) (In Chinese)

| Iterm | MeSH | Study number |
| --- | --- | --- |
| #1 | (题名或关键词=参附注射液 OR 摘要=参附注射液) AND (years:[1990 TO 2022]) | 3248 |
| #2 | (((((((((((((((题名或关键词=急性心肌梗死 OR 题名或关键词=acute myocardiac infarction) OR 题名或关键词=acute myocardial infarction) OR 题名或关键词=急性心肌硬死) OR 题名或关键词=心梗) OR 题名或关键词=急性心梗) OR 题名或关键词=急性心急梗死) OR 题名或关键词=心肌梗死) OR 题名或关键词=心肌梗塞) OR 题名或关键词=急性心机梗死) OR 题名或关键词=急性心肌梗死患者) OR 题名或关键词=急性心肌梗塞) OR 题名或关键词=老年急性心肌梗死) OR ((((((((((((摘要=急性心肌梗死 OR 摘要=acute myocardiac infarction) OR 摘要=acute myocardial infarction) OR 摘要=急性心肌硬死) OR 摘要=心梗) OR 摘要=急性心梗) OR 摘要=急性心急梗死) OR 摘要=心肌梗死) OR 摘要=心肌梗塞) OR 摘要=急性心机梗死) OR 摘要=急性心肌梗死患者) OR 摘要=急性心肌梗塞) OR 摘要=老年急性心肌梗死)) OR ((((((((((((题名或关键词=急性心肌梗死 OR 题名或关键词=acute myocardiac infarction) OR 题名或关键词=acute myocardial infarction) OR 题名或关键词=急性心肌硬死) OR 题名或关键词=心梗) OR 题名或关键词=急性心梗) OR 题名或关键词=急性心急梗死) OR 题名或关键词=心肌梗死) OR 题名或关键词=心肌梗塞) OR 题名或关键词=急性心机梗死) OR 题名或关键词=急性心肌梗死患者) OR 题名或关键词=急性心肌梗塞) OR 题名或关键词=老年急性心肌梗死)) OR ((((((((((((摘要=急性心肌梗死 OR 摘要=acute myocardiac infarction) OR 摘要=acute myocardial infarction) OR 摘要=急性心肌硬死) OR 摘要=心梗) OR 摘要=急性心梗) OR 摘要=急性心急梗死) OR 摘要=心肌梗死) OR 摘要=心肌梗塞) OR 摘要=急性心机梗死) OR 摘要=急性心肌梗死患者) OR 摘要=急性心肌梗塞) OR 摘要=老年急性心肌梗死)) AND (years:[1990 TO 2022]) | 142385 |
| #3 | ((((((((((((((((((((((((((题名或关键词=心力衰竭 OR 题名或关键词=cardiac failure) OR 题名或关键词=heart failure) OR 题名或关键词=慢性心衰竭) OR 题名或关键词=慢性心功能不全) OR 题名或关键词=慢性心力衰揭) OR 题名或关键词=急性充血性心力衰竭) OR 题名或关键词=慢性心脏功能衰竭) OR 题名或关键词=急性心衰) OR 题名或关键词=慢性充血性心衰) OR 题名或关键词=慢性心力衰竭) OR 题名或关键词=充血性心衰) OR 题名或关键词=充血心力衰竭) OR 题名或关键词=慢性心力衰) OR 题名或关键词=慢性心功能衰竭) OR 题名或关键词=充血性心力衰竭) OR 题名或关键词=慢性心衰) OR 题名或关键词=性心力衰竭) OR 题名或关键词=急性心功能衰竭) OR 题名或关键词=心功能不全) OR 题名或关键词=心衰) OR 题名或关键词=慢性充血性心力衰竭) OR 题名或关键词=急性心力衰竭) OR ((((((((((((((((((((((摘要=心力衰竭 OR 摘要=cardiac failure) OR 摘要=heart failure) OR 摘要=慢性心衰竭) OR 摘要=慢性心功能不全) OR 摘要=慢性心力衰揭) OR 摘要=急性充血性心力衰竭) OR 摘要=慢性心脏功能衰竭) OR 摘要=急性心衰) OR 摘要=慢性充血性心衰) OR 摘要=慢性心力衰竭) OR 摘要=充血性心衰) OR 摘要=充血心力衰竭) OR 摘要=慢性心力衰) OR 摘要=慢性心功能衰竭) OR 摘要=充血性心力衰竭) OR 摘要=慢性心衰) OR 摘要=性心力衰竭) OR 摘要=急性心功能衰竭) OR 摘要=心功能不全) OR 摘要=心衰) OR 摘要=慢性充血性心力衰竭) OR 摘要=急性心力衰竭)) OR ((((((((((((((((((((((题名或关键词=心力衰竭 OR 题名或关键词=cardiac failure) OR 题名或关键词=heart failure) OR 题名或关键词=慢性心衰竭) OR 题名或关键词=慢性心功能不全) OR 题名或关键词=慢性心力衰揭) OR 题名或关键词=急性充血性心力衰竭) OR 题名或关键词=慢性心脏功能衰竭) OR 题名或关键词=急性心衰) OR 题名或关键词=慢性充血性心衰) OR 题名或关键词=慢性心力衰竭) OR 题名或关键词=充血性心衰) OR 题名或关键词=充血心力衰竭) OR 题名或关键词=慢性心力衰) OR 题名或关键词=慢性心功能衰竭) OR 题名或关键词=充血性心力衰竭) OR 题名或关键词=慢性心衰) OR 题名或关键词=性心力衰竭) OR 题名或关键词=急性心功能衰竭) OR 题名或关键词=心功能不全) OR 题名或关键词=心衰) OR 题名或关键词=慢性充血性心力衰竭) OR 题名或关键词=急性心力衰竭)) OR ((((((((((((((((((((((摘要=心力衰竭 OR 摘要=cardiac failure) OR 摘要=heart failure) OR 摘要=慢性心衰竭) OR 摘要=慢性心功能不全) OR 摘要=慢性心力衰揭) OR 摘要=急性充血性心力衰竭) OR 摘要=慢性心脏功能衰竭) OR 摘要=急性心衰) OR 摘要=慢性充血性心衰) OR 摘要=慢性心力衰竭) OR 摘要=充血性心衰) OR 摘要=充血心力衰竭) OR 摘要=慢性心力衰) OR 摘要=慢性心功能衰竭) OR 摘要=充血性心力衰竭) OR 摘要=慢性心衰) OR 摘要=性心力衰竭) OR 摘要=急性心功能衰竭) OR 摘要=心功能不全) OR 摘要=心衰) OR 摘要=慢性充血性心力衰竭) OR 摘要=急性心力衰竭)) OR ((((((((((((((((((((((题名或关键词=心力衰竭 OR 题名或关键词=cardiac failure) OR 题名或关键词=heart failure) OR 题名或关键词=慢性心衰竭) OR 题名或关键词=慢性心功能不全) OR 题名或关键词=慢性心力衰揭) OR 题名或关键词=急性充血性心力衰竭) OR 题名或关键词=慢性心脏功能衰竭) OR 题名或关键词=急性心衰) OR 题名或关键词=慢性充血性心衰) OR 题名或关键词=慢性心力衰竭) OR 题名或关键词=充血性心衰) OR 题名或关键词=充血心力衰竭) OR 题名或关键词=慢性心力衰) OR 题名或关键词=慢性心功能衰竭) OR 题名或关键词=充血性心力衰竭) OR 题名或关键词=慢性心衰) OR 题名或关键词=性心力衰竭) OR 题名或关键词=急性心功能衰竭) OR 题名或关键词=心功能不全) OR 题名或关键词=心衰) OR 题名或关键词=慢性充血性心力衰竭) OR 题名或关键词=急性心力衰竭)) AND (years:[1990 TO 2022]) | 135793 |
| #4 | ((题名或关键词=RCT OR 题名或关键词=随机对照) OR (摘要=RCT OR 摘要=随机对照)) AND (years:[1990 TO 2022]) | 64029 |
| #5 | (((((((题名或关键词=西医治疗 OR 题名或关键词=western medical treatment) OR 题名或关键词=western medicine treatment) OR 题名或关键词=西药治疗) OR (((摘要=西医治疗 OR 摘要=western medical treatment) OR 摘要=western medicine treatment) OR 摘要=西药治疗)) OR (((((题名或关键词=常规治疗 OR 题名或关键词=conventional therapy) OR 题名或关键词=conventional treatment) OR 题名或关键词=routine therapy) OR 题名或关键词=routine treatment) OR 题名或关键词=常规处理)) OR (((((摘要=常规治疗 OR 摘要=conventional therapy) OR 摘要=conventional treatment) OR 摘要=routine therapy) OR 摘要=routine treatment) OR 摘要=常规处理)) OR ((((题名或关键词=药物治疗 OR 题名或关键词=drug therapy) OR 题名或关键词=drug treatment) OR 题名或关键词=medication) OR 题名或关键词=药物疗法)) AND (years:[1990 TO 2022]) | 85422 |
| #6 | #1 AND #2 AND #3 AND #4 AND #5 | 28 |
